# Analysis of gene expression in response to water deficit of chickpea (*Cicer arietinum *L.) varieties differing in drought tolerance

**DOI:** 10.1186/1471-2229-10-24

**Published:** 2010-02-09

**Authors:** Deepti Jain, Debasis Chattopadhyay

**Affiliations:** 1 National Institute of Plant Genome Research, Aruna Asaf Ali Marg, New Delhi-110067, India

## Abstract

**Background:**

Chickpea (*C. arietinum *L.) ranks third in food legume crop production in the world. However, drought poses a serious threat to chickpea production, and development of drought-resistant varieties is a necessity. Unfortunately, cultivated chickpea has a high morphological but narrow genetic diversity, and understanding the genetic processes of this plant is hindered by the fact that the chickpea genome has not yet been sequenced and its EST resources are limited. In this study, two chickpea varieties having contrasting levels of drought-tolerance were analyzed for differences in transcript profiling during drought stress treatment by withdrawal of irrigation at different time points. Transcript profiles of ESTs derived from subtractive cDNA libraries constructed with RNA from whole seedlings of both varieties were analyzed at different stages of stress treatment.

**Results:**

A series of comparisons of transcript abundance between two varieties at different time points were made. 319 unique ESTs available from different libraries were categorized into eleven clusters according to their comparative expression profiles. Expression analysis revealed that 70% of the ESTs were more than two fold abundant in the tolerant cultivar at any point of the stress treatment of which expression of 33% ESTs were more than two fold high even under the control condition. 53 ESTs that displayed very high fold relative expression in the tolerant variety were screened for further analysis. These ESTs were clustered in four groups according to their expression patterns.

**Conclusions:**

Annotation of the highly expressed ESTs in the tolerant cultivar predicted that most of them encoded proteins involved in cellular organization, protein metabolism, signal transduction, and transcription. Results from this study may help in targeting useful genes for improving drought tolerance in chickpea.

## Background

Drought continues to be one of the most significant environmental stresses as a result of continuous decrease in soil moisture content and increase in global temperature [1]. Rapid expansion of water-stressed areas necessitates improvement of crops with traits such as drought tolerance and adaptation, through conventional breeding and/or genetic manipulation. For cultivated crops like chickpea, where improvement through conventional breeding is difficult because of a narrow genetic base, comparative gene expression profiling is an alternate way to identify pathways and genes regulating the stress response [2]. Plants induce expression of a number of genes in response to water limitation. The early response at the cellular level results partly from cell damage, and corresponds partly to adaptive processes that initiate changes in the metabolism and structure of the cell that allows it to function under low water potential [3]. A wide range of techniques and strategies are being deployed these days to identify genes involved in stress responses [4]. But, while the advent of microarrays and protein profiling has generated a lot of information on gene expression during stress response, conventional gene-by-gene analysis is needed to validate these claims.

Most of the data on gene expression in plants in response to drought and other abiotic stresses has been generated using *Arabidopsis *[5-7]. However, in view of the wide genetic diversity that exists in the plant kingdom, this data may not hold true for other species. Therefore, individual crop-types should be studied to understand crop-specific responses to a particular stress. Among crop plants, cereals are the most studied with respect to gene expression because of their economic value and ample resources for research [8-17]. For example, a comparative gene expression study between a salt-tolerant and a salt-sensitive rice cultivar has shown that expression of genes related to protein synthesis and turnover were delayed in the sensitive variety and were perhaps responsible for the differential response [18]. However, a recent report suggested that salt-tolerance was due to the constitutive expression of some stress responsive genes that in the sensitive variety were inducible [17]. Transcriptional profiling of developing maize kernels in response to water deficit indicated that two classes of stress-responsive genes exist; one being specific to concurrent application of stress and another remains affected after transient stress [19]. A previous study from our group also indicated that the dehydration-induced expression of some genes in chickpea remain unaffected even after removal of dehydration stress and may lead to adaptation [20]. All these data point towards a hypothesis that a plant that is well adapted to stress has two basic mechanisms of stress-tolerance; constitutive expression of genes required for adaptation and quick expression of genes required to repair cellular damage and physiological reprogramming in adverse conditions. Comparative gene expression studies using cultivars with contrasting stress-tolerance features has become a useful tool to identify these two classes of genes.

In this study chickpea (*Cicer arietinum*), a popular food legume crop was used for analysis of gene expression under drought stress. Although chickpea is generally grown in relatively less irrigated lands and some cultivars adapt well to the water-limited environment [21], drought poses a serious threat to chickpea production causing 40-50% reduction of its yield potential [22]. Lack of adequate genetic and genomic resources impede progress of crop improvement in chickpea. In one study, a pulse microarray, containing about 750 cDNAs from chickpea, grass-pea and lentil, was used for the analysis of gene expression in response to water limitation, cold temperatures, and high salinity, in chickpea cultivars with contrasting stress-tolerance features [23]. Similarly, a database was generated from an EST library constructed by subtractive suppressive hybridization (SSH) of root tissue of two chickpea cultivars [24]. Comparative proteome maps of chickpea nucleus and cell wall also revealed differentially expressed proteins during water-deficit stress [25,26]. An exhaustive study on rapid dehydration-induced 26 bp SuperSAGE tags that were generated from root EST libraries of untreated and 6 h rapid dehydration-treated chickpea seedling has been reported. In addition, over 7000 UniTags having more than 2.7 fold abundance were identified in the dehydration libraries. Microarray analysis of 3000 of them exhibited about 80% congruency with the SuperSAGE data [27].

We have previously reported 101 ESTs of chickpea that were up-regulated more than 2 fold in response to rapid dehydration as compared to control conditions in the laboratory [20]. However, drought conditions in the field are quite different. Furthermore, transcriptional activation of a particular gene by drought might not be directly related to drought tolerance. In this study, the gene expression of a relatively drought-tolerant and a drought-sensitive chickpea cultivar were compared in response to progressive depletion of water. We have constructed SSH libraries from whole seedlings of the two cultivars at different stages of water depletion. A number of genes that express constitutively, as well as many that were induced quickly after application of stress in the tolerant cultivar, were identified. Annotation by homology search indicated that these genes are involved in cellular organization, protein metabolism, signal transduction and transcription.

## Results and Discussion

### Differential drought tolerance in two chickpea cultivars

**Figure 1:**
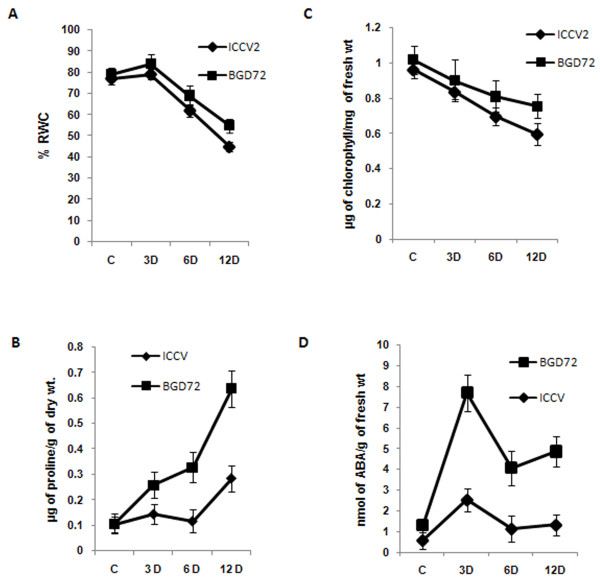
**Comparison of drought tolerance in two chickpea cultivars**. Comparative analysis of leaf relative water content (RWC) (A), proline (B), chlorophyll content (C), and ABA accumulation (D) between two (PUSABGD72, ICCV2) varieties of chickpea in a time dependent manner under drought stress. All experiments were done in triplicates, and average mean values were plotted against drought stress duration.

A comparison of drought tolerance between two cultivated chickpea varieties (*Cicer arietinum*, cv. PUSABGD72 and ICCV2) was conducted to establish their contrasting characters. The changes in leaf relative water content (RWC), chlorophyll content, abscisic acid (ABA) and proline were measured in seedlings grown for 12 d after germination before stopping irrigation (drought, DH stress) for different time points. RWC is a measure of stress-adaptation and accounts for osmotic adjustment, which is considered to be one of the most important mechanisms for adaptation to water-limited environment in plants. During the treatment both cultivars showed a little increase in RWC after 3 d (Figure 1A). This could either be due to increased transport of water from other compartments of the plant to the leaf in order to maintain turgor, or may have resulted from an osmotic adjustment due to increased synthesis of osmolytes. After this initial increase, leaf RWC of both the cultivars showed steep decrease up to the end of treatment (12 d). However, PUSABGD72 registered about 10% higher RWC than ICCV2 at the end-point (Figure 1A). Although the role of proline in stress tolerance is debatable, its accumulation is considered to be one of the indicators of adaptive response [28,29]. Both the cultivars showed greater proline content within 3 d of treatment and maintained the increase up to 12 d. However, proline accumulation in PUSABGD72 was more than two fold higher than in ICCV2 (Figure 1B). Chlorophyll content is considered to be the measure of rate of photosynthesis. This started decreasing with the initiation of DH in both the cultivars, but better maintenance in the rate of photosynthesis was displayed by PUSABGD72 throughout the course of stress treatment (Figure 1C). ABA acts as a key regulator of the dehydration response [30]. Most of the dehydration-inducible genes respond to treatment with exogenous ABA [31]. The course of ABA accumulation in both cultivars followed the same pattern, with PUSABGD72 showing constitutively higher ABA content than ICCV2. There was a sharp increase in the accumulation of ABA within 3 d indicating its involvement in early response to stress, whereas in the later period of treatment, ABA content was re-adjusted and maintained. Overall, ABA accumulation in PUSABGD72 throughout the treatment was 3 fold higher than in ICCV2 (Figure 1D). Taken together PUSABGD72 displayed a better tolerance to drought stress than ICCV2 with respect to the above assays.

### Cloning and sequencing of chickpea ESTs differentially expressed during drought stress

**Figure 2:**
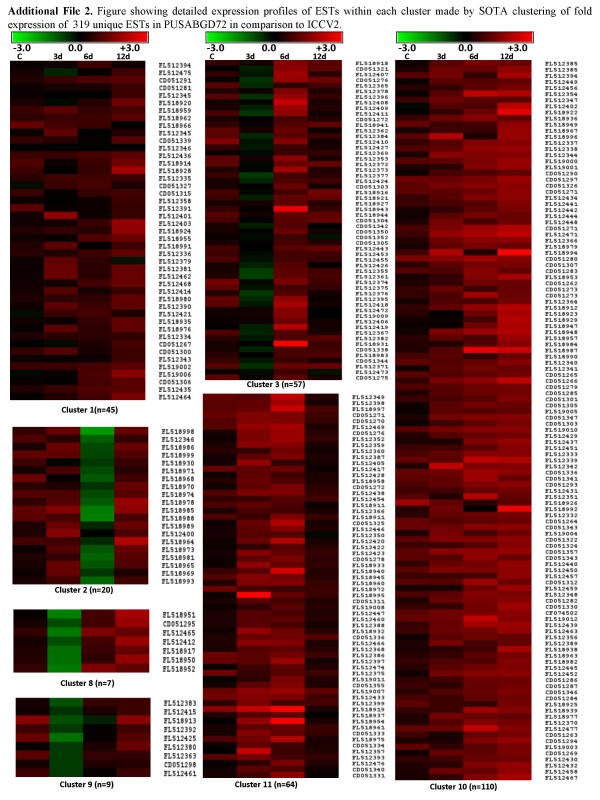
**Schematic representation of SSH libraries**. Schematic representation of five (four from this and one from another study [20]) subtractive cDNA libraries (SSH) prepared with chickpea seedlings.

**Figure 3:**
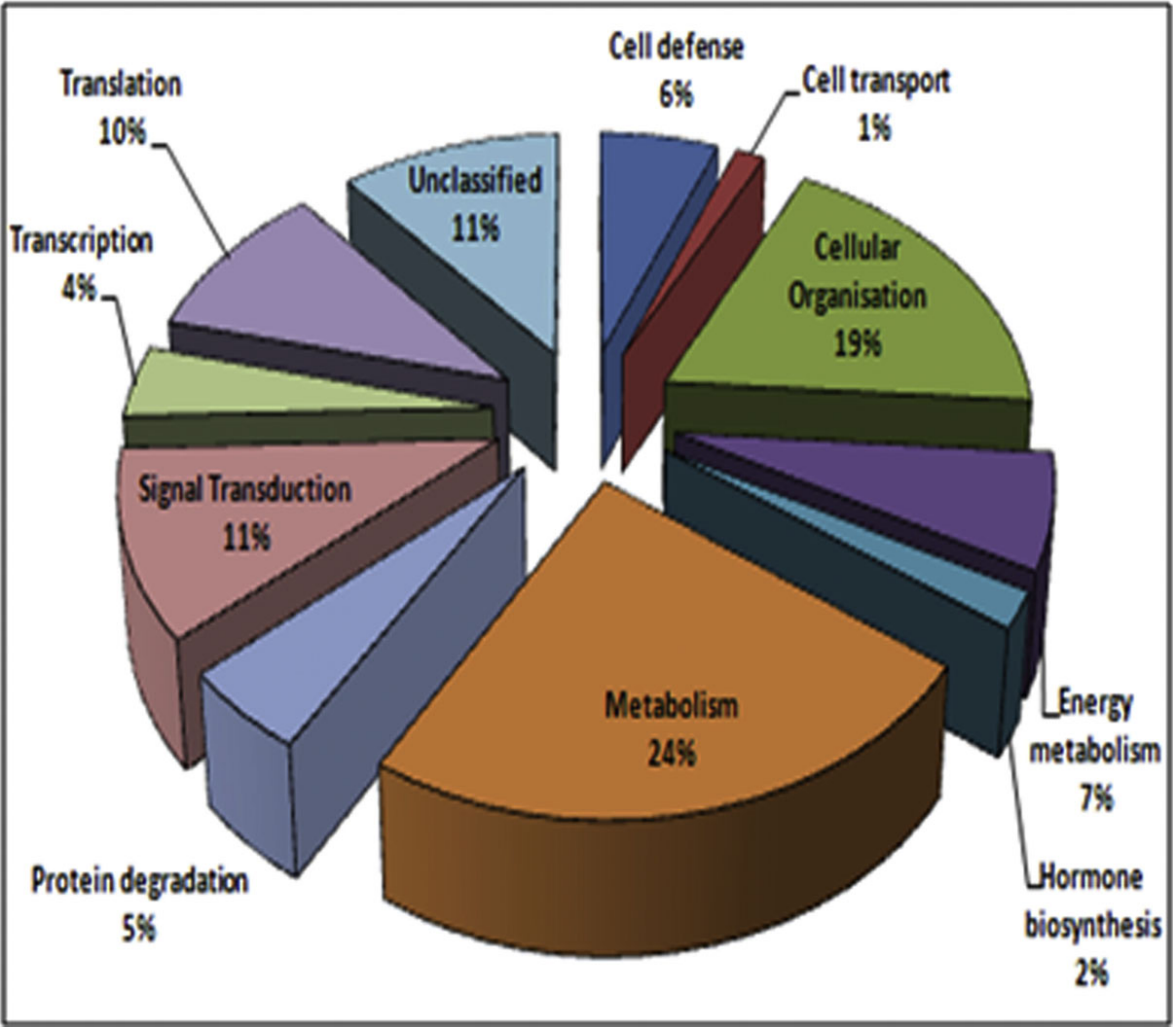
**Functional categorization of differentially expressed ESTs**. The identified ESTs were assigned with a putative function using BLASTX algorithm and were categorized with known or putative functional annotation. Detail information of each category is given in Additional File 1.

Plants perceive and respond to stress. Upon perception of stress, a signal is communicated to downstream components resulting in change of gene expression and thereby of proteins required for the initial damage-repair and physiological re-programming for better adaptation. Since physiological parameters studied under drought stress conditions indicated that PUSABGD72 was more drought-tolerant compared to ICCV2, we intended to identify the transcripts that were more abundant in the former. We used subtractive cDNA suppression hybridization (SSH) technology to clone these transcripts. SSH is widely used to screen differentially expressed genes because of its high efficiency in enriching low expressing genes and normalization of targeted fragments, Four subtracted cDNA libraries were constructed with poly (A^+^) RNA as described in Methods (Figure 2). 2700 randomly selected clones from all the libraries were single-pass sequenced and 319 high-quality unique ESTs were generated, which were deposited to GenBank. These were analyzed for putative functional classification by similarity search in the current GenBank database using the BLASTX algorithm. Of these, 312 ESTs showed significant similarity to known sequences, while the remaining 7 ESTs were deemed novel (Additional File 1). Further, 277 could be functionally categorized according to their BLASTX match and the remaining 35 ESTs of unknown function along with the 7 novel ESTs were kept under 'unclassified' category. The 277 functionally categorized ESTs represented genes involved in metabolism (24%), cellular organization (19%), protein metabolism (degradation and synthesis) (16%) and signal transduction (11%) cell defense (7%), cell transport (2%), energy metabolism (7%), hormone biosynthesis (3%), and transcription (5%). The 'unclassified' EST clones (as described above) accounted for 12% (Figure 3). A number of ESTs representing known stress-responsive proteins were present in abundance in the libraries, indicating their high expression in drought stressed seedlings. Among the most notable are the genes encoding β-amylase (6 clones), MIPS (11 clones), albumin (19 clones), polygalacturonase inhibiting protein (13 clones), 9-cis-epoxycarotenoid dioxygenase (7 clones), chaperons (like HSPs; 21clones), dehydrins (33 clones), proteases (35 clones), translation factors (43 clones) and transporters (29 clones).

### Comparative transcript profile of PUSABGD72 with respect to ICCV2

The expression of 319 unique ESTs obtained from the SSH libraries was analyzed by reverse-northern experiment as described previously [20]. PCR amplified ESTs were spotted in duplicate on nylon membranes in a 96-spot format. Chickpea *Actin *gene was spotted as a control for normalization and the kanamycin resistance gene, *NPTII *was used as the negative control for background subtraction. Radio-labeled first strand cDNA probes prepared using poly (A^+^) RNA isolated from control/stressed samples of PUSABGD72 or ICCV2 were used for hybridization and ESTs expressed differentially in the two cultivars were identified by the obtained differential hybridization intensities. Expression of each clone was tested in at least three independent drought stress experiments to confirm reproducibility. Expression ratio was calculated following the methods described in previous studies [5,20]. Signal intensity of each spot was normalized by subtracting the intensity of the negative control (*NPTII*). Fold expression was presented as the expression ratio (control/stressed) of PUSABGD72 to ICCV2 relative to the ratio of intensity of *Actin*. Genes showing ≥ 2 fold higher expression in PUSABGD72 at any time point in comparison to ICCV2 were considered as differentially expressed and studied further. Approximately 23%, 42.5%, 55.62% and 53.5% of the ESTs showed more than two-fold abundance in PUSABGD72 at control, 3 d, 6 d and 12 d DH conditions respectively. Relatively higher number of ESTs expressed differentially during DH treatment in PUSABGD72. 19.5% of all the ESTs showed ≥ 2 fold higher abundance in PUSABGD72 relative to that in ICCV2 at all the time points. ESTs expressing more in PUSABGD72 in comparison to ICCV2 at control condition naturally include drought-responsive and non-responsive genes.

**Figure 4:**
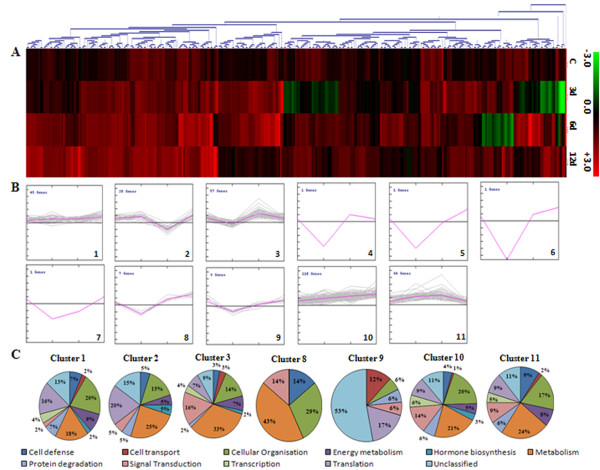
**Hierarchical clustering analysis of 319 unique genes based on their gene expression patterns in PUSABGD72 in comparison to ICCV2**. The 319 differentially expressed chickpea genes were distributed into 11 clusters based on their expression profiles. (A), the SOTA clustering tree. (B), expression profiles of SOTA clusters. The expression profile of each individual gene in the cluster is denoted by *grey line *and the mean expression profile is depicted by *pink line*. The number of genes in each cluster is given in the left upper corner and the cluster number is given in the right lower corner. (C), functional characterization of genes in each cluster. Detail information of genes within each cluster is elaborated in Additional File 2 and 3.

To achieve a comprehensive overview of relative expression profiles, 319 ESTs were clustered according to their relative expression patterns in PUSABGD72 in comparison to ICCV2 by the hierarchical clustering method using the correlation coefficient of average linkage of the log-transformed ratio [32,33]. SOTA clustering classified all the ESTs into 11 groups according to the distance of correlation (Figure 4). The data was taken in terms of fold expression of ESTs at control or DH stress in PUSABGD72 relative to that in ICCV2. The data sets were log-transformed to the base 2 to normalize the scale of expression and to reduce the noise. The clusters with n>6 were used to study the co-expression patterns of the genes. Detailed information on ESTs within each cluster is presented in Additional File 2 and 3. The ESTs belonging to cluster 1, 10 and 11 particularly were never found to be expressed less in PUSABGD72 as compared to ICCV2. ESTs of cluster 1 showed equivalent expressions in both the cultivars at control condition, however, expressed relatively higher in PUSABGD72 during DH treatment. Apart from the ESTs in the unclassified group, these ESTs are mainly involved in cellular organization, metabolism and protein translation category. Cluster 10 ESTs exhibited higher expression in PUSABGD72 at all the time points during the DH treatment. Genes related to cellular organization, metabolism and signal transduction mostly constitute this cluster. Seven transcription-related and eighteen protein metabolism-related ESTs are also included in this cluster that comprises nearly 36% of the total ESTs. Cluster 11 genes represented those that had higher expression in PUSABGD72 only at 3 d and at 6 d DH conditions, but were similar to ICCV2 at the later phase of stress. Important genes to mention in this cluster are several defense related genes such as polygalactouronase inhibitor proteins, MRP like ABC transporter and genes involved in sugar metabolism and photosynthesis. Interestingly, these three clusters included a lot of genes that showed homology with those encoding ribosomal proteins and translation elongation. This is in keeping with a previous study that also reported an early expression of genes involved in protein synthesis in a salt-tolerant rice variety in response to salt stress [8]. Genes involved in signal transduction showed a simple pattern of relative expression. Most of them, present in cluster 10 (16 ESTs) showed a steady higher abundance in PUSABGD72 at all time points. Interestingly, three ESTs representing a CBL-interacting protein kinase, a receptor-like kinase and a phosphoglycerate kinase showed higher relative expression in PUSABGD72 only at 6 d DH (cluster 3). Most of the ESTs that were more abundant in PUSABGD72 represented functions for cellular organization and metabolism. They displayed complex relative expression patterns probably because they were involved in different pathways. The ESTs that belong to the unclassified group showed no distinct clustering patterns, which may be due to their heterologous composition. Comparative transcriptome profiling suggested that PUSABGD72 possesses a different gene expression pattern from ICCV2 under drought stress.

It is already mentioned that about 23% of the ESTs (77 ESTs) showed ≥ 2 fold higher abundance in PUSABGD72 relative to that in ICCV2 at unstressed condition. Comparative transcript profiling revealed that 84% (65 ESTs) of these ESTs expressed more during the course of DH stress in PUSABGD72 in comparison to ICCV2. This result indicated that expression of most of the ESTs in this category is regulated by DH stress.

### Monitoring expression profiles of selected high expressing genes

More stringent criteria were applied to shortlist the genes that were showing drastic expression differences in the two cultivars. The genes showing ≥ 2 fold higher relative expression at the unstressed condition and ≥ 3 fold higher relative expression at any point of DH stress treatment in PUSABGD72 in comparison to ICCV2 were considered. This stringent parameter screened 49 genes (Additional File 4) from the 77 described above. Eight of these belonged to signal transduction category e.g. CBL-interacting protein kinase (CIPK) [FL512440], putative protein kinases [CD051343, CD051317], protein phosphatase 2C [CD051312], G-protein coupled receptor [CD051322], 14-3-3 protein homolog [FL512351]. Implication of SOS2-like protein kinases (CIPKs) in providing abiotic stress tolerance by activating the membrane-bound transporters is well documented [7,34-36]. Protein phosphatase 2C was shown to interact with SOS2 and mediate ABA-responsive signals [36,37,48]. Seven genes of transcription factor category mostly represented AP2-domain containing proteins. Members of the AP2/EREBP family of transcription factors, especially those that recognize drought-responsive element (DRE) in target promoters mediate distinct responses to abiotic stresses such as drought, salt and cold [38,39]. Another gene in this group putatively encoded a α-NAC transcription factor. NAC belongs to a family of proteins specific to plants and are found to play a role in a diverse set of developmental processes including formation and maintenance of shoot apical meristem and floral morphogenesis [40,41]. Overexpression of a NAC transcription factor in *Arabidopsis *up-regulated several stress-responsive genes in the transgenic plants, and thereby conferred drought tolerance [42]. Zinc finger proteins [FL512439] are ubiquitous; some of them were shown to provide tolerance against abiotic stresses [43,44]. Six ESTs represented well-known stress responsive genes encoding ABA-responsive protein [FL512397], stress activated protein [FL512411], salt tolerance proteins [FL512396, FL518936], dehydration-induced protein [FL512471]. High expression of ten genes under cellular organization category was well understood as they putatively encoded LEAs and dehydrins. Higher accumulation of dehydrin mRNA transcript in drought tolerant sunflower was associated with cellular turgor maintenance under drought stress [45]. Dehydrin, LEA and proline rich proteins are thought to provide stability to other proteins in osmotic stress [3]. High relative expression of six genes related to protein metabolism corroborates the results of a previous study with rice cultivars [8].

**Figure 5:**
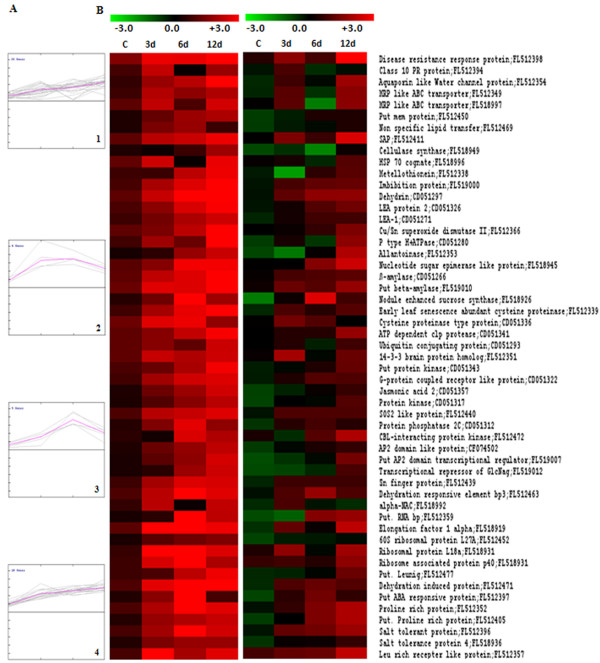
**Hierarchical clustering analysis of 53 selected genes based on their gene expression patterns**. Analysis of expression profiles of 53 ESTs (Additional File 4) in PUSABGD72 and ICCV2 with and without water-deficit stress. (A), ESTs were grouped into four clusters based on their expression profiles in PUSABGD72. The expression profile of individual gene in the cluster is denoted by *grey line *and the mean expression profile is depicted by *pink line*. The number of genes in each cluster is given in the left upper corner and the cluster number is given in the right lower corner. Detail information of genes within each cluster is elaborated in Additional File 5 and 6. (B), the comparative expression profiles of 53 ESTs in PUSABGD72 and ICCV2.

**Figure 6:**
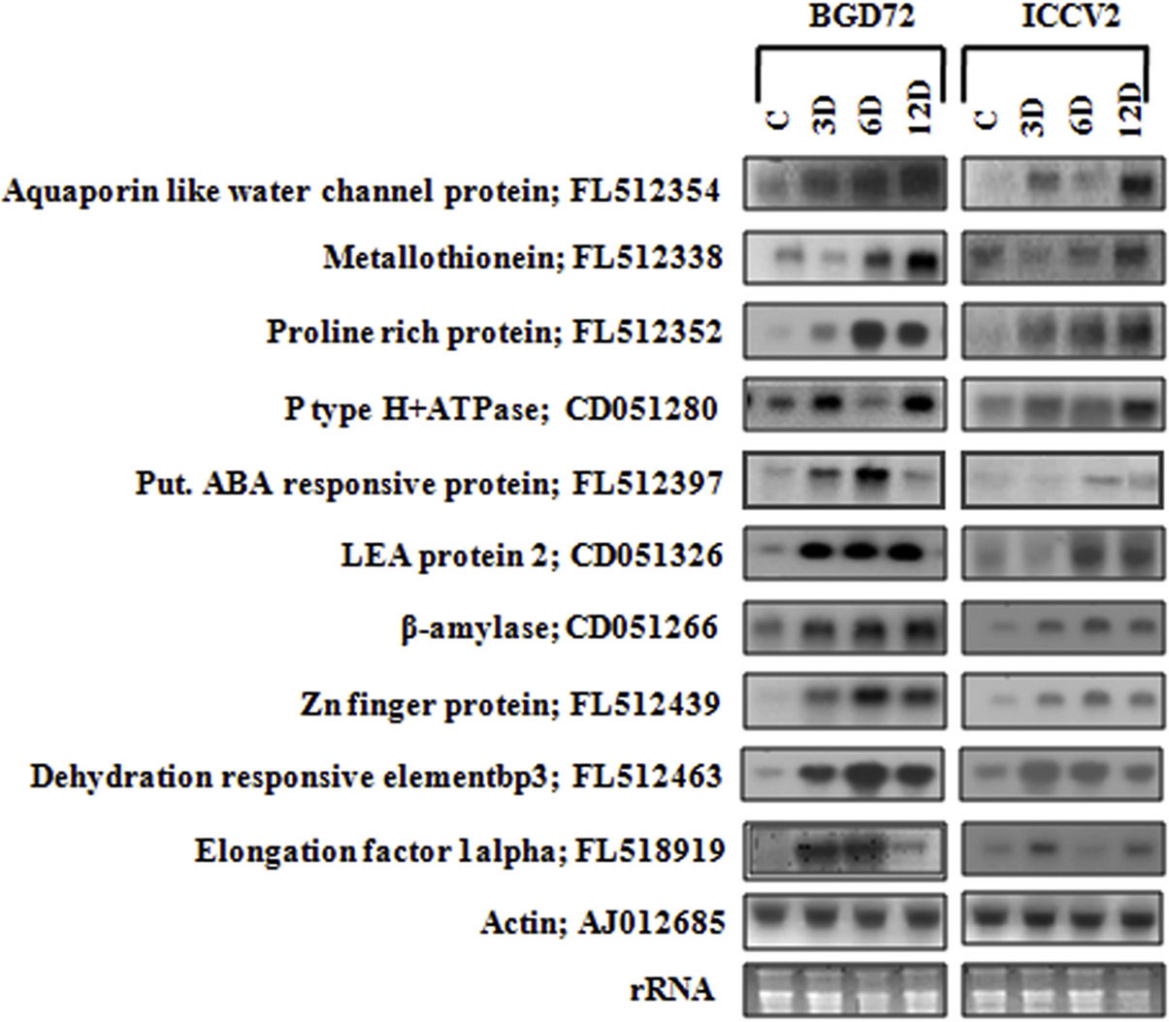
**Northern analysis of selected stress responsive genes**. Northern analysis showing expression of ten selected stress responsive genes [Aquaporin like water channel protein; GenBank: FL512354, Metallothionein; GenBank: FL512338, Proline rich protein; GenBank: FL512352, P type H^+^ATPase; GenBank: CD051280, Putative ABA response protein; GenBank: FL512397, LEA protein 2; GenBank: CD051326, β-amylase; GenBank: CD051266, Zn finger protein; GenBank: FL512439, Dehydration responsive element bp3; GenBank: FL512463 and Elongation factor 1 alpha; GenBank: FL518919] in PUSABGD72 and ICCV2. 20 μg of total RNA isolated from control/stressed seedlings of PUSABGD72/ICCV2 were separated in formaldehyde denaturing gel, transferred to nylon membrane and probed with α^32^P-dCTP labeled amplified cDNA fragments corresponding to indicated EST clones. An amplified product of chickpea *Actin *cDNA was used as an internal control and 28S ribosomal RNA was shown as loading control. Time points in days (d) are indicated.

Overexpression of superoxide dismutase has been implicated in free radical detoxification and suggested to have a major role in defending the mangrove species against severe abiotic stresses [46]. Four ESTs were identified on the basis of early expression upon DH treatment in PUSABGD72. One of them was a CBL-interacting protein kinase [FL512472], two represented ribosomal proteins [FL518931, FL518954] and one was a leucine rich repeat protein [FL512357] (Additional File 4). Absolute expression of all these 53 ESTs in the two contrasting cultivars was compared and presented in Figure 5. These 53 ESTs can be clustered in four groups according to their expression in the tolerant cultivar PUSABGD72 (Additional File 5 and 6). 44 out of 53 high expressing ESTs belonged to clusters 1 and 4. The mean curves of these two clusters registered a steady increase in gene expression from unstressed condition to the end of DH stress treatment, although, there was a basic difference between these two clusters. Average expression intensity of the cluster 4 genes was much higher than that of the cluster 1 genes and there was uniformity in the expression of the cluster 4 genes. Two ESTs [FL512394 and FL518992] of the cluster 1 displayed a rapid induction at 3 d DH; but their expression went down bellow their basal expression level at 6 d DH time point, however, upregulated again at 12 d. Their expression was checked in three different biological samples in triplicates by qReal Time-PCR to avoid any error (Additional File 7). One of these two ESTs encoded PR-10 protein. Although, the PR proteins are implicated in cellular defense as they express under pathogen attack abiotic stresses like drought and salinity also induce their expression. Stable expression of a pea PR-10 in *Brassica *enhanced its germination and growth in presence of sodium chloride [47]. The other EST coded for a NAC transcription factor. Interestingly, most of the ESTs belonging to signal transduction category exhibited a steady increase in expression under DH stress condition from their basal level. Only two of them, one encoding a protein phosphatase 2C (CD051312) and the other, CBL-interacting protein kinase (FL512472) showed sudden high expression at 6 d DH and then reduced at 12 d DH. NAF domains of CIPKs were shown to interact with phosphatase 2C (ABI1 and ABI2) [36,48]. Similar expression pattern of these ESTs correlates with their mutual interaction. Except NAC, the other ESTs encoding transcription factors in this cluster expressed steadily higher than their basal level. Five genes of cluster 3 that showed sudden high expression at 6 d DH condition mostly represent proteins of unknown function. Another EST of this cluster encoded a putative RNA binding protein and suddenly expressed 20 fold high at 6 d DH in PUSABGD72. Expression of this EST in ICCV2 also followed a similar pattern, but with a much lower absolute value (Figure 5). Role of a specific glycine-rich RNA binding protein in regulation of stomata and thereby in abiotic stress response is already reported [49]. The cluster 2 genes, that showed a rapid high fold of expression at 3 d DH and maintained that up to 6 d DH belong to protein metabolism category. Three of them were related to protein synthesis (elongation factor, ribosomal proteins), and exhibited 15-fold high expression early in the DH treatment. Another represented a factor involved in protein degradation and expressed about 4 fold higher than its basal expression level at 3 d DH. Fold expression of these genes in the sensitive cultivar ICCV2 at the same time point was comparatively much lower. Interestingly, two ESTs representing elongation factor 1 alpha (FL518919) and ribosomal protein L18a (FL518931) also showed early induction in ICCV2, but their absolute levels of expression were much lower than that in PUSABGD72 (Figure 5). To validate the results obtained by reverse northern analysis, RNA accumulation of ten ESTs (FL512354, FL512338, FL512352, CD051280, FL512397, CD051326, CD051266, FL512439, FL512463 and FL518919) was monitored in both the cultivars by northern analysis (Figure 6). Overall, the result of northern blot analysis was in agreement with the expression data obtained by reverse-northern analysis.

We recently reported the functional validation of two chickpea genes corresponding to two differentially expressed ESTs described in this study; one (FL512440) codes for a CBL-interacting proteins kinase (CaCIPK6; GenBank: DQ239702) and another (FL512348) for a zinc finger protein (CaZF; GenBank: EU513298). Expression of *CaCIPK6 *in tobacco and *Arabidopsis *conferred improved tolerance against high concentration of sodium chloride and mannitol [50]. Ectopic expression of *CaZF *improved germination efficiency of transgenic tobacco in presence of high salinity [51].

## Conclusions

To date, a limited number of studies on drought stress-mediated gene expression in chickpea have been reported. In this study we described an analysis of gene expression in chickpea in response to drought stress and intended to carry out a comparative transcript profiling between the contrasting chickpea varieties. We focused on a set of transcripts that exhibited higher abundance in a drought-tolerant cultivar in comparison to a drought-sensitive one. We took suppressive subtractive hybridization (SSH) approach to construct the EST libraries because chickpea EST resources are limited. We applied water-deficit stress by withdrawal of irrigation for three different periods. This allowed us to perform a series of comparison of transcript abundance between and within the chickpea varieties at different time points of stress treatment. Comparative expression profiles categorized the ESTs in 11 clusters according to their relative expression patterns. 53 ESTs were identified on the basis of their very high fold of relative expression in the tolerant variety. High fold of abundance of these transcripts in the tolerant variety might be just correlative and establishment of any relation between this transcript abundance and drought-tolerance in chickpea is beyond the scope of the experiments performed in this study. We also do not intend to comment that the mechanism of drought-tolerance in chickpea is limited to only transcriptional upregulation of some genes. The purpose of this study was to compare two contrasting chickpea varieties and to generate a resource to initiate gene-by-gene analysis for drought-tolerance mechanism.

The differential expression pattern of the transcripts observed might be applicable only to these two particular chickpea varieties used in this study, although the genes identified on the basis of differential expression patterns corroborate with results from some of the similar studies on other plants [8,52]. In this study, a stress condition close to field drought was applied. Field drought is a slow process and the plants go through an adaptive process in contrast to the drastic condition of rapid dehydration. Furthermore, due to narrow genetic diversity among the cultivated legume varieties the genes that express co-incidentally due to DH stress may be common in both the varieties and, therefore, might not have been highlighted in a comparative gene expression analysis. These might be the reasons for less number of differentially expressed transcripts detected in our study in comparison to that in the SAGE analysis [27].

Temperate grain legumes such as pea, fava bean and lentil share similar gene arrangement with chickpea [53]. It is, therefore, expected that this data will benefit the study of the similar grain legume crops. Since the genes that experience subtle changes in expression in DH stress might not have been detected due to the stringent method of construction of SSH cDNA library, much robust experimentation involving oligonucleotide-based microarrays supported by enough EST resources is required for clear understanding.

## Methods

### Plant materials and stress treatments

Chickpea (*Cicer arietinum *L. cv PUSABGD72 and ICCV2) seeds (provided by IARI, New Delhi, India and ICRISAT, Hyderabad, India respectively) were grown in 3 L pots with composite soil (peat compost to vermiculite, 1:1) for 12 d after germination at 22 ± 2°C and 50 ± 5% relative humidity with a photoperiod of 12 h. Both the cultivars were grown in the same pot so that they were exposed to the same soil moisture content. The pots were irrigated with 200 ml water everyday. For drought treatment, soil-grown 12 day-old plants were subjected to progressive drought by withholding water for 3, 6, and 12 d respectively. In this period the soil moisture content decreased from approximately 50% to approximately 15% at the end of 12 d. As a control, some plants were kept under the same condition for the same period with watering. Drought stressed plants were harvested at the same time of the day to avoid diurnal changes; immediately frozen in liquid nitrogen and stored at -80°C before RNA isolation. Relative water contents of the leaves were measured at the corresponding time points following standard method [26].

### RNA isolation and construction of subtracted cDNA library

Total RNA was isolated from the harvested seedlings by using TRIzol Reagent (Life Technologies, Rockville, MD), and poly (A^+^) RNA was purified by mRNA isolation kit (Roche Applied Science, Manheim, Germany). Subtracted cDNA library was constructed by using CLONTECH PCR-Select cDNA subtraction kit (CLONTECH Laboratories, Palo Alto, CA) following the method provided by the manufacturer. In brief, tester (C/3 d/6 d/12 d drought PUSABGD72) and driver (C/3 d/6 d/12 d drought ICCV2) double stranded cDNAs were prepared from poly (A^+^) RNA (2 μg each) samples. The cDNAs were digested with *RsaI *and then ligated to different adaptors present in the kit. Two rounds of hybridization and PCR amplification (Advantage 2 PCR kit, CLONTECH) were performed to normalize and enrich the differentially expressed cDNAs. The forward subtracted and enriched DNA fragments were directly cloned into T/A cloning vector (pGEM-T Easy Vector Systems, Promega, USA). Competent cells of *E. coli *DH5α were prepared by CaCl_2 _method and transformed with the ligation mix and plated on Luria-Bertani (LB) agar plates containing ampicillin (selection marker), IPTG, and X-gal for blue-white selection [54]. All the recombinant clones were pooled to establish the subtracted cDNA library.

### Amplification of cDNA inserts

The cDNA insert of individual clones of the subtracted cDNA library were amplified by polymerase chain reaction (PCR) (Perkin-Elmer GeneAmp PCR System 9600) using M13 forward and M13 reverse primers in a 50 μL reaction with thermo-cycling condition: an initial denaturation at 94°C for 10 min, followed by 30 cycles of 94°C for 30 s, 60°C for 1 min, 72°C for 2 min and a final extension at 72°C for 10 min. The PCR products were analyzed by agarose gel electrophoresis for insert size, amplification quality and quantity. The positive clones were then selected for sequencing.

### Sequence analysis

The selected positive clones were all single-pass sequenced using Big Dye Terminator kit version 3.0 (Applied Biosystems, Foster City, CA) and analyzed with the ABI Prizm 3700 DNA analyzer. The base-calling of the chromatogram files was performed automatically by PHRED processing [55] with sequence quality value of 20. Vector sequences were removed by CROSS_MATCH http://www.genome.washington.edu, and the polyA tails were trimmed off by Trimest of EMBOSS application http://www.emboss.org. Finally, high quality sequences were selected with base-calling error of ≤ 1% and reads of ≥ 200 bp. Each edited EST was searched against non-redundant protein database of NCBI http://www.ncbi.nlm.nih.gov using BLASTX. The default BLAST parameters were used. Putative functions to the ESTs were assigned based on the results of BLASTX searches. All cDNA fragments are registered in NCBI EST database. Unique ESTs were selected for further analysis.

### cDNA Macroarray preparation

Purified PCR products were denatured by adding an equal volume of 0.6 M NaOH. Equal volume of each denatured PCR product (≈ 100 ng) of ≥ 200 bp of size was spotted on two Hybond N membranes (Amersham Pharmacia Biotech, Uppsala) using dot-blot apparatus in 96 format to make two identical arrays. In addition, PCR products of chickpea *Actin *cDNA [GenBank: AJ012685] and Neomycin phophotransferase (*NPTII*) gene from the vector pCAMBIA 1305.1 [GenBank: AF354045] were spotted as internal and negative controls respectively to normalize the signals of two replicate blots corresponding to stressed/unstressed chickpea cultivars and to subtract the background intensity respectively. The membranes were neutralized with neutralization buffer (0.5 M Tris-HCl, pH 7.4; 1.5 M NaCl) for 3 min, washed with 2× SSC, and cross-linked by UV cross linker (Stratagene, La Jolla, CA).

### Probe preparation and reverse northern hybridization

cDNAs were labeled with α^32^P-dCTP in the first-strand reverse transcription of mRNA. One microgram of mRNA was labeled in a 20 μL reaction volume containing 1× reaction buffer, 2 μg of 5'-(dT)_30_VN-3' (V = A/G/C and N = A/G/C/T) primer, 2.5 mM dATP, dTTP, dGTP, 0.02 mM dCTP, 5 μL of α^32^P-dCTP (10 μCi/μL; 3000 μCi/mmol), and 200 units of reverse transcriptase (Superscript II, Life Technologies, Grand Islands, NY). After incubation at 42°C for 1 h, mRNA was removed by incubating with RNaseH (Life Technologies, Grand Islands, NY) at 37°C for 20 min. Radiolabeled cDNAs were cleaned by Sephadex G-25 (Amersham-Pharmacia Biotech) and suspended in hybridization buffer (7% SDS, 0.3 M Sod-phosphate pH 7.4, 1 mM EDTA, 10 μg of sonicated salmon sperm DNA). Nylon membranes were pre-hybridized with the same buffer for 3 h at 65°C and hybridized with denatured cDNA probes at the same condition for 24 h. The membranes were washed three times (10 min each) with washing buffer (2XSSC, 0.1% SDS, 65°C). The replicate membranes were then exposed to same storage phosphor screen (Amersham Biosciences, Piscataway, NJ) for 2 d. Images of the membranes were acquired by scanning with a Typhoon 9210 scanner (Amersham Biosciences).

### Data analysis for DNA-array

Data analysis was performed using ImageQuant software (Molecular Dynamics, Sunnyvale, CA). The radioactive intensity of each spot was quantified as volume value. The local background value was subtracted resulting in the subtracted volume values (sVol). *Actin *cDNA was used as the internal control and its subtracted volume value was designated as sRef. Normalization among all images was performed by dividing sVol of each spot by the sRef value within the same image resulting in a normalized volume value (nVol) for each spot. nVol values of each EST spot in two identical arrays were compared. Three independent experiments were conducted to assess the reproducibility of the macroarray analysis. Data presented for the expression profile analysis is an average of three independent experiments. Expression profiles of the stress inducible genes were analyzed by the hierarchical SOTA (Self-organizing tree algorithm) clustering on the log-transformed-fold induction expression values across four time points by using MultiExperiment Viewer (MEV) Software (The Institute for Genome Research; http://rana.lbl.gov/EisenSoftware.htm) [32].

### Northern analysis

Total RNA was isolated from seedlings at different stress time points, separated by electrophoresis in denaturing formaldehyde 1.2% (w/v) agarose gels and transferred to Hybond-N^+ ^nylon membrane (GE Healthcare, Buckinghamshire, UK) following the method mentioned in Sambrook *et al*. (2001) [54]. PCR-amplified individual cDNA fragment (with primers corresponding to adaptor 1 and 2R used for preparing SSH library) was purified from agarose gel and used as a probe. Probes were labeled with α^32^P-dCTP using Megaprime DNA labeling system (GE Healthcare) and purified through Sephadex G-25. Northern hybridization, washing and scanning were performed and band-intensity was calculated by following the procedure described above for nylon membrane array.

### Determination of relative water content (RWC)

Chickpea leaf tissues were collected and immediately weighed [fresh weight, FW]. The tissues were rehydrated in water for 24 h until fully turgid, surface-dried, reweighed [turgid weight, TW] followed by oven drying at 80°C for 48 h, and reweighed [dry weight, DW]. The RWC was calculated by the following formula: RWC (%) = (FW-DW/TW-DW) ×100 [56]. The experiment was carried out in triplicates.

### Estimation of abscisic acid, proline, and chlorophyll

ABA content of chickpea seedlings with or without stress was measured according to Setter *et al*. (2001) [57]. Lyophilized seedlings were crushed in chilled 80% methanol. The extracts were fractionated by C18 reverse-phase chromatography, and the ABA-content was assayed by enzyme linked immunosorbant assay (ELISA). The ABA-content is expressed as microgram of ABA per gram of dry weight. Free Proline content was measured as described by Bates et al. (1973) [58]. The tissues were homogenized in 3% aqueous sulfosalicylic acid. The homogenate was centrifuged at 9000× *g *and the supernatant was collected. The reaction mixture consisted of 2 ml of supernatant, 2 ml of acid-ninhydrin, and 2 ml of glacial acetic acid, which was boiled at 100°C for 1 h. After termination of the reaction on ice, the reaction mixture was extracted with 4 ml of toluene, and the absorbance was read at 520 nm. The assays were done in triplicates using corrected weight calculated for the actual moisture content of tissue at each time point. For chlorophyll estimation, tissues harvested at different time points were ground in 80% chilled acetone. The supernatant was taken and absorbance was read at 663 nm, 645 nm and 480 nm and calculated according to Lichtenthaler *et al*. (2001) [59]. The experiments were done in triplicates using corrected tissue weights calculated for actual moisture content of the tissue at the respective time points.

## Authors' contributions

DJ carried out the experimental studies, data acquisition, analysis and interpretation of data. DC has designed the work and drafted the manuscript and revised it critically. All authors read and approved the final manuscript.

## Supplementary Material

Additional file 1 Functional categorization of ESTs generated by subtracted cDNA libraries. Click here for file

Additional file 2 Figure showing detail expression profiles of ESTs within each cluster made by SOTA clustering of fold expression of 319 unique ESTs in PUSABGD72 in comparison to ICCV2. Click here for file

Additional file 3 Table showing detail cluster information made by SOTA clustering of fold expression of ESTs in PUSABGD72 in comparison to ICCV2. Click here for file

Additional file 4 **Transcript expression profiles of selected 53 genes in PUSABGD72 seedlings.** Transcript expression analysis in response to drought stress at different time points with the fold-expression values. [Standard deviations (SD ±) are calculated from three different experiments. The transcripts are listed according to their putative functions]. Click here for file

Additional file 5 Figure showing detail expression profiles of ESTs within each cluster made by SOTA clustering of fold expression of 53 high expressing ESTs in PUSABGD72. Click here for file

Additional file 6 Table showing detail clustering of 53 high expressing ESTs according to their expression pattern in PUSABGD72. Click here for file

Additional file 7 **Quantitative Real time-PCR analysis in different biological samples of PUSABGD72 and ICCV2 showing normalized fold induction of two ESTs (FL12394, FL518992) upon drought treatment. **The experiment was done in triplicates by taking *Actin *as an internal control. Click here for file
