# Supplementary material for: Analysis of gene expression in response to water deficit of chickpea (Cicer arietinum L.) varieties differing in drought tolerance
Source: BMC Plant Biol. 2010 Feb 9;10:24. doi: 10.1186/1471-2229-10-24 (PMC2831037; doi:10.1186/1471-2229-10-24)
Supplement: Additional file 1 — Functional categorization of ESTs generated by subtracted cDNA libraries. [file 1471-2229-10-24-S1.DOC]

**Additional File 1**: Functional categorization of ESTs generated by subtracted cDNA libraries.

| **GenBank Match** | **Putative fuction** | **E-value** | **Accession No.** |
| --- | --- | --- | --- |
| **Cell Defense** |  |  |  |
| AAC49797.1 | MRP like ABC transporter | 8E-77 | FL512349 |
| CAA10134.1 | Basic blue Cu protein | 7E-21 | FL512383 |
| CAA31760.1 | Disease resistance response protein | 1E-43 | FL512398 |
| CAD59565.1 | PDR like ABC transporter | 3E-77 | FL512404 |
| BAC66711.1 | Put. Cold shock protein | 0.021 | FL512475 |
| AAQ93011.1 | Put chloroplast FtsH proteinase | 8E-26 | FL518918 |
| BAE71245.1 | FtsH like protein PftF precursor | 2E-88 | FL518951 |
| ABO61512.1 | Leu rich recepter like protein | 4.E-05 | FL512357 |
| AAB32504.1 | Class 10 PR protein | 1E-23 | FL512394 |
| BAF00814.1 | MRP like ABC transporter | 7E-43 | FL518997 |
| ABG34276.1 | Polygalacturonase like protein | 8E-52 | FL518998 |
| BAA29056.1 | Polygalacturonase inhibiting protein | 7E-47 | CD051271 |
| AAK59442.1 | Chitinase family 19 | 1E-34 | CD051291 |
| BAB01963.1 | Polygalacturonase inhibiting protein | 3E-10 | CD051270 |
| BAB16429.1 | Elicitor inducible gene | 8E-11 | FL512456 |
| **Cell Transport** |  |  |  |
| CAB61749.1 | Aquaporin | 7E-29 | FL512407 |
| CAD56222.1 | Aquaporin like Water channel protein | 4E-51 | FL512354 |
| AAB60858.1 | Vacuolor assembly protein | 2E-43 | CD051281 |
| CAB81061.1 | Kinesin like protein | 2E-49 | CD051276 |
| **Cellular Organisation** |  |  |  |
| CAA05771.1 | Lipid transfer protein precursor | 4E-53 | CD051321 |
| CAA05771 | Lipid transfer protein | 1E-53 | FL512385 |
| AAF26451 | Non specific lipid transfer | 9E-14 | FL512469 |
| CAA78515.1 | Dehydrin | 6E-12 | FL512347 |
| AAO65979.1 | Seed protein | 3E-18 | FL512360 |
| CAB71135.1 | Put. Imbibition protein | 1E-99 | FL512365 |
| AAX86047.1 | α –Tubulin | 7E-77 | FL512378 |
| CAA10131.1 | Chalcone synthetase | 1E-24 | FL512402 |
| AAF18411.1 | Integral mem. Protein | 8E-107 | FL512408 |
| ABD32895.1 | HSP70 | 9E-105 | FL512415 |
| BAE71209.1 | Importin | 3E-82 | FL512417 |
| CAA04767.1 | Ripening induced protein | 5E-32 | FL512428 |
| BAD38204.1 | HVA-22 homolog | 3.E-06 | CD051272 |
| AAA33671.1 | Probable HSP | 4E-15 | FL518913 |
| BAB09641.1 | Ceramidase family protein | 1E-26 | FL518915 |
| AAN31874.1 | Anion exchange protein | 3E-92 | FL518920 |
| ABD32352.1 | HSP | 3E-56 | FL518941 |
| AAM61627.1 | Put ER lumen protein retaining receptor | 5E-83 | FL518946 |
| AAR23312.1 | Cellulose synthase | 1E-108 | FL518949 |
| ABO47740.1 | α- tubuline | 8E-55 | FL518958 |
| CAB75430.1 | Put 16KD mem protein | 3E-40 | FL518959 |
| ABD32895.1 | 70KD HSP | 2E-93 | FL518962 |
| CAB45653.1 | Put tonoplast intrinsic protein | 1E-26 | FL518966 |
| CAA05979.1 | Adenine ntd translocator | 4E-80 | FL518967 |
| AAL38353.1 | HSP | 3E-48 | FL518986 |
| ABD32895.1 | HSP 70 cognate | 6E-77 | FL518996 |
| CAA10192.1 | Glycine rich protein | 2E-09 | FL512344 |
| CAB71135.1 | Imbibition protein | 2E-38 | FL519000 |
| AAG15412.1 | Seed maturation protein | 5E-29 | FL519001 |
| CAB53509.1 | Histone 2A | 2E-22 | CD051290 |
| AAN77521.1 | Dehydrin | 3E-25 | CD051297 |
| CAA12027 | LEA protein 2 | 6E-37 | CD051326 |
| CAA12026.1 | LEA-1 | 6E-16 | CD051271 |
| AAM61711.1 | Prolyl-4-hydroxylase | e-100 | CD051295 |
| AAC33276.1 | Fiber protein 1 | 5E-33 | CD051339 |
| AAN77521.1 | Dehydrin1 | 4E-38 | FL512434 |
| AAM61107.1 | Probable nitrate transporter | 3E-12 | FL512436 |
| BAB16458.1 | Nitrate transporter | 5E-10 | FL512438 |
| CAA48210 | Triose Phos translocator | 4E-52 | FL512441 |
| AAB71830.1 | Annexin | 1E-15 | FL512442 |
| AAK53759.1 | Probable K+ transporter | 1E-25 | FL512444 |
| AAK76516.1 | Aconitase family | 4E-70 | FL512448 |
| AAF63170.1 | Endomembrane protein 70 | 3E-71 | FL512454 |
| CAA10127.1 | Nucleolar protein | 2E-25 | FL512465 |
| AAM08004.1 | Put mem protein | 1E-20 | FL512450 |
| AAD30230.1 | Membrane protein (CDC50 family protein) | 1E-54 | FL512474 |
| CAA66038.1 | Proline rich protein | 3E-15 | FL512352 |
| CAB81548.2 | Put. Proline rich protein | 4E-79 | FL512405 |
| **Energy metabolism** |  |  |  |
| CAA65008.1 | Metallothionein-like protein 1 (MT-1) | 2E-26 | FL512338 |
| AAF04584.1 | Type 1 metallothionein | 1E-44 | FL512409 |
| CAA10132.1 | Superoxide dismutase | 3E-82 | FL512362 |
| CAA39819.1 | Cu/Zn superoxide dismutase II | 4E-62 | FL512366 |
| CAB71128.2 | Cationic peroxidase | 1E-82 | FL512384 |
| AAY56795.1 | Vacuolor ATP synthetase | 8E-79 | FL512392 |
| ABO20848.1 | Monooxygenase | 9E-48 | FL512410 |
| CAA06156.1 | Cyt. P450 monooxygenase | 1E-31 | FL518911 |
| CAA59444 | Catalase | 3.E-09 | FL512416 |
| BAA10929.1 | Unsp. Monooxygenase | 2E-42 | FL512427 |
| AABO1223 | Put dehydrogenase E1 beta | 8E-45 | FL518914 |
| ABN08957.1 | H+-transporting two-sector ATPase | 2E-58 | FL518930 |
| ABA01324.1 | Epoxide hydrolase | 7E-16 | FL518928 |
| AAW51769.1 | Catalase fragment | 5E-14 | FL518979 |
| BAD26579.1 | Cyt P450 like_TBP | 1E-45 | FL518994 |
| CAA08855.1 | Copper amine oxidase | 2E-84 | FL512335 |
| CAC29436.1 | P type H+ATPase | 1E-11 | CD051280 |
| CAA45098 | Thioredoxin F type, chloroplast precursor | 0.007 | CD051307 |
| AAM61418.1 | Mitochondrial uncoupling protein | 2E-18 | CD051283 |
| AAG14962.1 | Cyt P450 dependent monooxygenase | 8E-62 | CD051325 |
| BAB33250.1 | NADH dehydrogenase ND1 | 4E-27 | CD051327 |
| CAB78385.1 | Put-3-isopropylmalate dehydrogenase | 1.E+00 | FL512446 |
| **Hormone biosynthesis** |  |  |  |
| CAA53730.1 | Lipoxygenase | 2E-44 | FL512369 |
| ABO77438.1 | Methionine adenosyl transferase | 3E-46 | FL518953 |
| AAT40304.1 | S-adenosylmethionine synthetase (*Medicago*) | 3E-62 | FL518971 |
| AAA81377.1 | SAM synthetase (*Arabidopsis*) | 2E-22 | CD051262 |
| CAC43237.1 | Lipoxygenase | 2E-57 | CD051273 |
| BAC10549.1 | Nine cis epoxycarotenoid dioxygenase | 4E-52 | CD051315 |
| **Metabolism** |  |  |  |
| AAA33642 | Fructose-bisphosphate aldolase 1 | 3E-97 | FL512350 |
| AAR29343.1 | Allantoinase | 4E-31 | FL512353 |
| CAB10455.1 | Lipase | 9E-25 | FL512358 |
| ABM01871.1 | Chloroplast rubisco activase | 7E-56 | FL512364 |
| AAB81011.1 | Asn synthetase | 6E-69 | FL512372 |
| AAD27878.1 | Chlorophyll a/b binding protein | 1E-58 | FL512373 |
| ABI94075.1 | Chloroplast rubisco activase small protein isoform | 8E-30 | FL512377 |
| CAB10455.1 | Triacylglycerol lipase like protein | 9E-24 | FL512391 |
| BAC76729.1 | α – amylase | 4E-23 | FL512401 |
| CAC84547.1 | Dicarboxylate/tricarboxylate carrier | 1E-99 | FL512403 |
| AAD01737.1 | GDP mannose pyrophosphorylase | 2E-17 | FL512412 |
| ABP49577.1 | Microsomal omega-6-desaturase | 2E-103 | FL512413 |
| AAN75219.1 | Chloroplast translocon | 2E-68 | FL512420 |
| ABQ88337.1| | β – cobalamine synthase | 2E-44 | FL512422 |
| BAE07181.1 | Met. Synthase | 2E-60 | FL512423 |
| AAK65960.1 | Sucrose synthase | 5E-23 | FL512424 |
| AAB06756.2 | 1-L-myoinositol-1-P-synthetase | 6E-12 | CD051303 |
| AAM61665.1 | Leuco-anthocynidine dioxygenase | 5E-36 | CD051278 |
| CAH59405.1 | Light harvesting protein | 1E-72 | FL518912 |
| ABW21688.1 | Enolase, isoform 1 | 1E-96 | FL518916 |
| ACC59198.1 | 1-acyl-sn-glycerol-3-Phos acyltransferase | 4E-70 | FL518917 |
| AAQ84168.1 | 1-deoxy-o-xylulose-5-Phos reductoisomerase | 6E-89 | FL518921 |
| AAM28620.1 | adenosine monophosphate binding protein | 7E-63 | FL518923 |
| CAA04512.1 | Second sucrose synthase | 7E-79 | FL518924 |
| CAA36396.1 | Glyceraldehydes-3-Phos dehydrogenase | 1E-56 | FL518927 |
| ACC63885.1 | Caffeic acid-o-methyltransferase | 4E-67 | FL518929 |
| CAA76854.1 | Ketol acid reductoisomerase | 7E-37 | FL518933 |
| AAK64167.1 | Methionin synthase | 6E-83 | FL518940 |
| CAA84494.1 | α- 1,4-glucan phosphorylase | 1E-42 | FL518943 |
| CAA42443.1 | P-protein | 9E-59 | FL518944 |
| AAN15627.1 | Nucleotide sugar epimerase like protein | 2E-92 | FL518945 |
| AAP83930.1 | Rubisco activase b form precursor | 6E-17 | FL518947 |
| AAA33652.1 | Carbonic anhydrase | 3E-77 | FL518948 |
| AAB01223.1 | Pyruvate dehydrogenase E1 b | 6E-63 | FL518950 |
| AAM63830.1 | Put PS-I reaction | 2E-23 | FL518955 |
| AAT85058.1 | Put C type cyt synthase | 2E-42 | FL518957 |
| AAG52429.1 | Put amino peptidase | 1E-55 | FL518960 |
| CAB50768.1 | Cyt P450 | 9E-37 | FL518968 |
| AAB46611.1 | Asparate aminotransferase | 5E-61 | FL518970 |
| AAB99755.1 | Malate dehydrogenase | 4.E-06 | FL518972 |
| AAF98217.1 | Mannose – 6 – Phos isomerase | 3E-51 | FL518974 |
| AAN17423.1 | P-protein like protein | 3E-21 | FL518978 |
| ABB20913.1 | Rubisco activase | 7E-78 | FL518984 |
| AAN75219.1 | Rubisco activase (small isoform) | 4E-53 | FL518985 |
| AAB00860.1 | Microsomal omega-6-desaturase | 7E-92 | FL518987 |
| ABB29955.1 | Hydroxyacyl glutathione hydrolase | 2E-28 | FL518990 |
| BAA33879.1 | α-amylase | 6E-68 | FL518991 |
| CAA10290.1 | Rubisco small subunit | 4E-25 | FL518995 |
| AAM65487.1 | Chl a/b bp | 2E-25 | FL512336 |
| CAA63482.1 | Glycolate oxidase | 1E-74 | FL512340 |
| CAA06819.1 | Cysteine synthase | 1E-51 | FL512341 |
| BAB10198.1 | Alcohol dehydrogenase | 4E-12 | CD051265 |
| AAL67089.1 | β-amylase | 1E-89 | CD051266 |
| AAK27718.1 | ADP glucose pyrophosphorylase | 1E-33 | CD051279 |
| BAB08397.1 | Phosphoribosylanthranilate transferase like protein | 2E-17 | CD051285 |
| CAC07424.1 | Cinnamoyl CoA reductase | 2E-58 | CD051301 |
| AAK15160.1 | Put apyrase | 8E-48 | CD051304 |
| CAB78780.1 | Trehalose-6-Phos synthase homolog | 2E-25 | CD051305 |
| AAN15946.1 | Rubisco activase(small isoform) | 2E-12 | CD051311 |
| AAD25783.1 | Aldehyde dehydrogenase family | 1E-22 | FL519005 |
| AAM91301.1 | Phosphoglucomutase | 1E-34 | CD051347 |
| AAB99632.1 | Phophonopyruvate decarboxylase like protein | 2.E-04 | FL519008 |
| AAG09205.1 | Trans-cinnamate-4-monooxygenase | e-125 | CD051342 |
| CAA11857.1 | Delta-8-sphingolipid desaturase | 4.E-08 | CD051350 |
| AAF79428.1 | Long chain fatty acid condensing enzyme | e-111 | CD051352 |
| CAA89019.1 | Cobalamine independent methionine synthase | 4E-12 | CD051358 |
| AAL67089.1 | Put β- amylase | 2E-90 | FL519010 |
| CAB78780.1 | Trehalose-6-phosphate synthase like protein | 2E-25 | CD051305 |
| AAL37169.1 | Put chloroplast targeted b amylase | 2E-26 | FL512429 |
| AAD01804.1 | Lipase (class 3) family | 3E-14 | FL512437 |
| CAA06339.1 | UDP galactose-4-epimerase | 1E-34 | FL512443 |
| AAD02832.1 | Raffinose synthase | 7E-38 | FL512447 |
| AAF26084.1 | Put alkaline neutral invertase | 1E-66 | FL512451 |
| BAC01214.1 | Fructose-1,6-bisphosphatase | 6E-65 | FL512453 |
| AAO38524.1 | Asparagines synthetase | 5E-66 | FL512455 |
| AAL37169.1 | Glycosyl hydrolase family 14 | 3E-89 | FL512460 |
| BAB40340.1 | Probable 12 oxophytodienoate reductase | 7E-26 | FL512470 |
| AAC28107.1 | Nodule enhanced sucrose synthase | 4E-32 | FL518926 |
| **Protein degradation** |  |  |  |
| ABH08753.1 | Ubiquitin | 3E-63 | FL512379 |
| CAA08906.1 | Cysteine proteinase | 5E-49 | FL512381 |
| CAB88363.1 | Prolyl-peptidyl isomerase | 1E-47 | FL512388 |
| ABH08753.1 | Polyubiquitin | 3E-52 | FL512426 |
| ABR25718.1 | Monoubiquitin | 4E-28 | FL518932 |
| EAA21327.1 | Putative senescence associated protein | 2E-13 | FL518988 |
| ABD32628.1 | Thioprotease | 2E-72 | FL512333 |
| ABF18679.1 | Early leaf senescence abundant cysteine proteinase | 1E-72 | FL512339 |
| CAA08906.1 | Cysteine proteinase | 2E-86 | FL512342 |
| BAA88898.1 | Cysteine proteinase type protein | 1E-52 | CD051336 |
| BAB08738.1 | ATP dependent clp protease | 8E-73 | CD051341 |
| CAA51821.1 | Ubiquitin conjugating protein | 9E-56 | CD051293 |
| AAO43306.1 | Putative polyubiquitin | 2E-29 | FL512431 |
| AAA34123.1 | Hexameric polyubiquitin | 4E-27 | FL512462 |
| AAM64530.1 | Ubiquitin homolog | 3E-57 | FL512466 |
| **Signal Transduction** |  |  |  |
| ABQ95992.1 | 14-3-3 brain protein homolog | 3E-56 | FL512351 |
| CAC43238.1 | Calcium binding protein | 1E-20 | FL512355 |
| AAM62466.1 | Stress related protein | 4.E-04 | FL512361 |
| AAM63746.1 | ADP ribosylation factor like protein | 7E-70 | FL512368 |
| CAA88841.1 | Phosphoglycerate kinase | 5E-86 | FL512374 |
| AAS65786.1 | Protein kinase family (arabi) | 3E-36 | FL512375 |
| BAB10839.1 | Receptor like protein kinase | 5E-48 | FL512376 |
| AAD10151.2 | Put WD40 repeat protein | E-118 | FL512386 |
| CAL25342.1 | Putative Zinc binding protein | 2E-84 | FL512395 |
| AAL47552.1 | IAA-AA hydrolase | 7E-78 | FL512418 |
| CAA48210.1 | Phosphate translocator | 3E-69 | FL512425 |
| AAK50348.1 | CBL-interacting protein kinase | 6E-23 | FL512472 |
| AAG50535.1 | Put. Ser-thr protein kinase | 8E-26 | CD347670 |
| AAS65786.1 | Protein kinase family protein | 9E-37 | FL512375 |
| AAC62851.1 | Photolyase | 2E-48 | FL518952 |
| AAF18411.1 | Integral membrane protein | 4E-108 | FL518989 |
| BAD35220.1 | Put nucleolar GTP bp | 2E-24 | FL512332 |
| AAL47352.1 | WD repeat protein like protein | 4E-26 | CD051264 |
| AAM65034.1 | Put protein kinase | 3E-34 | CD051343 |
| AAD17804.1 | Nodule enhanced protein phosphatase | 1E-84 | FL519004 |
| AAM91135.1 | G-protein coupled receptor like protein | 5E-63 | CD051322 |
| AAM62611.1 | ADP ribosylation factor like protein | 2.E-09 | CD051324 |
| AAF04915.1 | Jasmonic acid 2 | 2E-19 | CD051357 |
| CAA67554.1 | Protein kinase (CIPK25) | 8.E-05 | CD051317 |
| CAA49171.1 | Omnipotent suppressor protein | 1E-30 | FL519009 |
| AAD17804.1 | Nodule enhanced phophorus protein | 1.E-07 | FL519011 |
| AAM83095.1 | SOS2 like protein (CIPK6) | 1E-43 | FL512440 |
| AAF69681.1 | High affinity Fe^+2^-Pb^+2^ permease | 2E-12 | FL512457 |
| CAB90633.1 | Protein phosphatase 2C | 5E-48 | CD051312 |
| CAA49512.1 | Glycerol kinase related | 2E-56 | FL512459 |
| BAA33803.1 | Chloroplast phosphoglycerate kinase | 7E-58 | FL512468 |
| **Transcription** |  |  |  |
| AAQ10954.1 | Zn finger protein | 1E-23 | FL512348 |
| CAD59768.1 | Putative reverse transcriptase | 2E-09 | FL512380 |
| CAE45592.1 | Transcription factor BTF3 | 2E-43 | FL512406 |
| AAZ14831.1 | AP2 transcription factor | 8E-22 | FL512414 |
| ABH02865.1 | Transcription factor Myb-1 | 9E-33 | FL512419 |
| BAB09451.1 | Histone acyl transferase | 2E-59 | FL518980 |
| AAN31856.1 | RNA helicase | 1E-21 | CD051282 |
| AAM47901.1 | RAP2.6 | 4E-17 | CD051355 |
| CAB96991.1 | Put Zn finger protein | 4E-22 | CD051330 |
| AAC49772.1 | AP2 domain like protein | 4E-17 | CF074502 |
| AAC49770.1 | Put AP2 domain transcriptional regulator | 9.E-09 | FL519007 |
| CAC92868.1 | Transcriptional repressor of GlcNag | 2E-46 | FL519012 |
| AAL61938.1 | Aspartyl-tRNA synthetase | 3E-46 | FL512433 |
| AAD26942.1 | Zn finger protein | 5E-16 | FL512439 |
| AAO13360.1 | Dehydration responsive element bp3 | 2E-19 | FL512463 |
| AAL66951.1 | α-NAC | 2E-09 | FL518992 |
| AAM66970.1 | Put. RNA bp | 5E-23 | FL512359 |
| ABB87134.1 | RNA bp | 8E-41 | FL512337 |
| BAE71244.1 | RNA bp | 5E-29 | FL518922 |
| AAM66970.1 | RNA bp cp29 protein | 9E-24 | FL512387 |
| **Translation** |  |  |  |
| AAN74635.1 | DEAD box RNA helicase | 3E-115 | FL512356 |
| CAI48073.1 | 60S ribosomal protein L37a | 4E-46 | FL512367 |
| AAZ32899.1 | Elongation factor 2 | 1E-75 | FL512382 |
| AAF27938.1 | Translation initiation factor 5A | 1E-14 | FL512389 |
| AAN15375.1 | Translation initiation factor eIF-2 | 1E-43 | FL512390 |
| AAM63913.1 | 40s ribosomal protein S7 homolog | 1E-17 | FL512399 |
| AAZ32899.1 | Elongation factor 2 | 2E-28 | FL512400 |
| CAA74893.1 | Translation elongation factor Tu | 7E-39 | FL512421 |
| CAA06245.1 | Elongation factor 1 alpha | 1E-52 | FL518919 |
| BAD09700.1 | Put ribosomal protein large subunit | 1E-83 | FL518935 |
| AAK25759.1 | Ribosomal protein L18a | 2E-34 | FL518931 |
| ABK63942.1 | Ribosomal protein L3 | 3E-42 | FL518934 |
| ABK63942.1 | 60S ribosomal protein L3 | 2E-48 | FL518937 |
| AAL09401.1 | Ribosomal protein L17 | 1E-61 | FL518938 |
| AAQ22726.1 | 40S ribosomal protein S25 | 8E-29 | FL518942 |
| CAA07226.1 | Ribosome associated protein p40 | 4E-83 | FL518954 |
| AAB81972.1 | Ribosomal protein S14 | 5E-41 | FL518961 |
| CAG47084.1 | 40S ribosomal protein S9 like | 3E-12 | FL518963 |
| CAA39950.1 | Ribosomal protein L11 | 5E-46 | FL518964 |
| ABF97261.1| | Put 40S ribosomal protein S3 | 4E-67 | FL518973 |
| ACF06499.1 | Put 40S ribosomal protein | 2E-63 | FL518976 |
| AAM65734.1 | 60s ribosomal protein L13a | 6E-71 | FL518981 |
| ACF06499.1 | 40S ribosomal protein | 1E-21 | FL518982 |
| BAA96366.1 | 40S ribosomal protein S13 | 4E-29 | FL512445 |
| AAM62795.1 | 60S ribosomal protein L27A | 6E-19 | FL512452 |
| CAD56219.1 | Ribosomal protein S3a | 2E-101 | FL512334 |
| AAD28753.1 | 60S ribosomal protein L37A | 1E-46 | CD051286 |
| BAA20879.1 | Eukaryotic translation initiation factor 5A-1 | 3E-59 | CD051287 |
| AAC14469.1 | 40S ribosomal protein S11 | 2E-32 | CD051346 |
| AAL79739.1| | Ribosomal protein | 4E-16 | CD051267 |
| AAN31818.1 | Ribosomal protein S15 | 2E-58 | CD051284 |
| CAA11705.1 | Elongation factor | 1E-25 | CD051300 |
| AAM67061.1 | Ribosomal protein S2 | 1E-50 | CD051333 |
| AAM63913.1 | 40S ribosomal protein S7 homolog | 4E-17 | CD051338 |
| **Unclassified** |  |  |  |
| AAS21370.1 | PB-1 domain containing protein | 2.E+00 | FL518925 |
| ACF06596.1 | Callus protein P23 | 6E-37 | FL518939 |
| AAP21292.1 | Armadillo b catenin repeat | E-29 | FL518965 |
| CAB81288.1 | DER2.2 | 3E-41 | FL518969 |
| ABK95544.1 | Unknown protein | 5E-26 | FL518975 |
| CAB79503.1 | Put KH domain protein | 4E-44 | FL518977 |
| ABQ41951.1 | SOUL protein | 8E-26 | FL518983 |
| ABO61376.1 | Serine hydroxymethyltransferase | 1E-39 | FL518993 |
| CAA66108.1 | Specific tissue protein | 2E-110 | FL512343 |
| CAC04249.1 | PPF-1 protein | 2E-48 | CD051344 |
| AAL06916.1 | Expressed protein | 3E-08 | CD051334 |
| ABO61512.1 | Put ABA responsive protein | 1E-16 | FL512397 |
| BAE19944.1 | UV opsin | 1E+00 | FL512363 |
| BAB09624.1 | Put. Protein (Arabi.) | 4E-33 | FL512370 |
| CAN81117.1 | Unknown protein (Arabi) | 9E-70 | FL512371 |
| AAN13040.1 | Put CCR-4 associated factor | 7E-22 | FL512393 |
| AAF33786.1 | Cold induced alfalfa gene | 1.E+00 | FL512473 |
| CAA11429.1 | Zwille protein shoot meristem | 3.E-05 | FL512476 |
| CAF18246.1 | Put. Leunig | 4E-17 | FL512477 |
| AAM67211.1 | Serine rich protein | 4E-16 | CD051340 |
| AAK00390.1 | Phi – 1 like protein | 1E-51 | CD051263 |
| AAL87341.1 | LIN 1 protein | 2E-30 | FL519002 |
| AAF33786.1 | Cold induced alfalfa gene | 9E-14 | CD051294 |
| AAO22640.1 | Put protein | 6.E-07 | FL519003 |
| AAM62421.1 | Drm3 | 5E-17 | CD051331 |
| AAK00390.1 | Put phi-1 protein | 4E-16 | FL519006 |
| AAC64888.1 | Hyp protein | 6E-42 | CD051269 |
| BAB62576.1 | Put protein | 8E-40 | CD051275 |
| AAK59423.1 | Putative protein | 4E-21 | CD051298 |
| AAM64945.1 | PDI like protein | 9.E-07 | CD051306 |
| AAL29690.1 | Profiling | 3E-55 | FL512430 |
| AAM34266.1 | VTC2 | 2E-80 | FL512432 |
| BAA33810.1 | Phi-1 | 3.E-08 | FL512435 |
| AAM14063.1 | Put cullin | 2E-32 | FL512458 |
| AAM12036.1 | Anther specific protein | 3E-23 | FL512461 |
| AAK98750.1 | Transposase of Tn10 | 1E-86 | FL512464 |
| BAB96814.1 | Zwille protein | E-05 | FL512467 |
| ACA04850.1 | SAP | 2E-40 | FL512411 |
| BAB33421.1 | Put SAP | 7E-27 | FL518999 |
| AAF75749.1 | Dehydration induced protein | 6E-28 | FL512471 |
| CAC85245.1 | Salt tolerant protein | 1E-42 | FL512396 |
| CAC85245.1 | Salt tolerance protein 4 | 4E-17 | FL518936 |
