# Supplementary material for: Analysis of gene expression in response to water deficit of chickpea (Cicer arietinum L.) varieties differing in drought tolerance
Source: BMC Plant Biol. 2010 Feb 9;10:24. doi: 10.1186/1471-2229-10-24 (PMC2831037; doi:10.1186/1471-2229-10-24)
Supplement: Additional file 2 — Figure showing detail expression profiles of ESTs within each cluster made by SOTA clustering of fold expression of 319 unique ESTs in PUSABGD72 in comparison to ICCV2. [file 1471-2229-10-24-S2.PPT]

## Slide 1
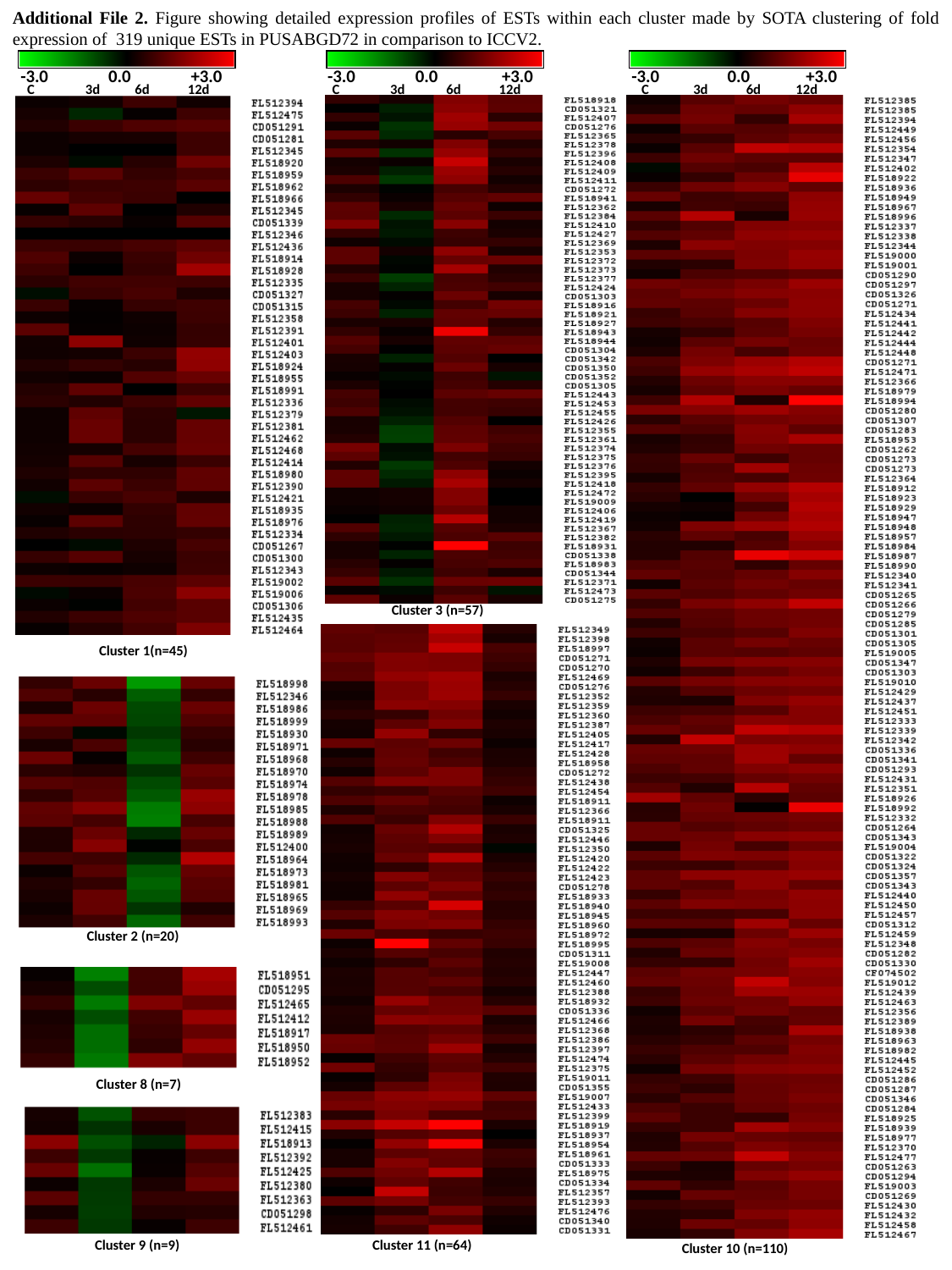

Additional File 2. Figure showing detailed expression profiles of ESTs within each cluster made by SOTA clustering of fold expression of 319 unique ESTs in PUSABGD72 in comparison to ICCV2.
-3.0 0.0 +3.0
-3.0 0.0 +3.0
-3.0 0.0 +3.0
C 3d 6d 12d
C 3d 6d 12d
C 3d 6d 12d
Cluster 3 (n=57)
Cluster 1(n=45)
Cluster 2 (n=20)
Cluster 8 (n=7)
Cluster 9 (n=9)
Cluster 11 (n=64)
Cluster 10 (n=110)
