# Supplementary material for: Analysis of gene expression in response to water deficit of chickpea (Cicer arietinum L.) varieties differing in drought tolerance
Source: BMC Plant Biol. 2010 Feb 9;10:24. doi: 10.1186/1471-2229-10-24 (PMC2831037; doi:10.1186/1471-2229-10-24)
Supplement: Additional file 3 — Table showing detail cluster information made by SOTA clustering of fold expression of ESTs in PUSABGD72 in comparison to ICCV2. [file 1471-2229-10-24-S3.DOC]

**Additional File 3**: Table showing detailed cluster information made by SOTA clustering of fold expression of ESTs in PUSABGD72 in comparison to ICCV2.

| **Annotation** | **Classification** | **Acc. No.** | **Log_2_(ctrl)** | **Log_2_ (3d)** | **Log_2_ (6d)** | **Log_2_ (12d)** |
| --- | --- | --- | --- | --- | --- | --- |
| **Cluster 1** | | | | | | |
| Class 10 PR protein | Cell defense | FL512394 | 0.151 | 0.227 | 0.731 | 0.222 |
| Put. Cold shock protein | Cell defense | FL512475 | 0.275 | 0.454 | 0.084 | 0.743 |
| Chitinase family 19 | Cell defense | CD051291 | 0.41 | 0.705 | 0.934 | 1.057 |
| Vacuolor assembly protein | Cell transport | CD051281 | 0.176 | 0.287 | 0.299 | 0.736 |
| Anion exchange protein | Cellular Organisation | FL518920 | 0.343 | -0.134 | 0.469 | 1.316 |
| Put 16KD mem protein | Cellular Organisation | FL518959 | 0.703 | 1.077 | 0.619 | 0.84 |
| 70KD HSP | Cellular Organisation | FL518962 | 0.546 | 0.696 | 0.736 | 1.064 |
| Put tonoplast intrinsic protein | Cellular Organisation | FL518966 | 1.208 | 0.807 | 0.7 | 0.021 |
| Fiber protein 1 | Cellular Organisation | CD051339 | 0.642 | 0.485 | 0.124 | 1.005 |
| Probable nitrate transporter | Cellular Organisation | FL512436 | 0.697 | 0.687 | 0.848 | 1.091 |
| Put dehydrogenase E1 beta | Energy metabolism | FL518914 | 0.935 | 0.153 | 0.69 | 1.329 |
| Epoxide hydrolase | Energy metabolism | FL518928 | 0.738 | 0 | 0.617 | 1.902 |
| Cu amine oxidase | Energy metabolism | FL512335 | 0.526 | 0.766 | 0.766 | 0.74 |
| NADH dehydrogenase subunit | Energy metabolism | CD051327 | 0.163 | 0.678 | 0.848 | 0.361 |
| Nine cis epoxycarotenoid dioxygenase | Hormone biosynthesis | CD051315 | 0.678 | 0.084 | 0.642 | 0.743 |
| Lipase | Metabolism | FL512358 | 0.172 | 0.07 | 0.189 | 0.722 |
| Triacylglycerol lipase like protein | Metabolism | FL512391 | 1.079 | 0.084 | 0.163 | 0.626 |
| α – amylase | Metabolism | FL512401 | 0.214 | 1.669 | 0.189 | 0.645 |
| Dicarb/tricarboxy carrier | Metabolism | FL512403 | 0.204 | 0.251 | 0.705 | 1.736 |
| Second sucrose synthase | Metabolism | FL518924 | 0.394 | 0.623 | 0.469 | 1.702 |
| Put PS-I reaction | Metabolism | FL518955 | 0.214 | 0.151 | 0.972 | 1.238 |
| α-amylase | Metabolism | FL518991 | 0.433 | 1.118 | 0.017 | 0.719 |
| Chl a/b bp | Metabolism | FL512336 | 0.526 | 0.401 | 0.74 | 1.16 |
| Polyubiquitin | Protein degradation | FL512379 | 0.176 | 1.131 | 0.536 | 0.264 |
| Cys. Proteinase | Protein degradation | FL512381 | 0.184 | 1.245 | 0.506 | 1.201 |
| Hexameric polyubiquitin | Protein degradation | FL512462 | 0.184 | 1.245 | 0.506 | 1.201 |
| Phosphoglycerate kinase | Signal Transduction | FL512468 | 0.214 | 0.263 | 0.816 | 1.238 |
| AP2 transcription factor | Transcription | FL512414 | 0.252 | 1.014 | 0.287 | 0.7 |
| Put HAT | Transcription | FL518980 | 0.365 | 0.575 | 0.574 | 1.151 |
| Translation initiation factor e/F-2 | Translation | FL512390 | 0.214 | 1.084 | 0.696 | 1.134 |
| Translation elongation factor | Translation | FL512421 | 0.163 | 0.678 | 0.848 | 0.361 |
| Put ribosomal protein large subunit | Translation | FL518935 | 0.234 | 0.151 | 0.607 | 1.16 |
| Put 40S ribosomal protein | Translation | FL518976 | 0.111 | 1.084 | 0.548 | 1.175 |
| Ribosomal protein S3a | Translation | FL512334 | 0.379 | 0.516 | 0.536 | 0.623 |
| Ribosomal protein | Translation | CD051267 | 0.009 | 0.136 | 0.367 | 0.84 |
| Elongation factor | Translation | CD051300 | 0.642 | 1.014 | 0.287 | 0.7 |
| Specific tissue protein | Unclassified | FL512343 | 0.172 | 0.124 | 0.189 | 0.722 |
| LIN 1 protein | Unclassified | FL519002 | 0.697 | 0.687 | 0.848 | 1.091 |
| Put phi-1 protein | Unclassified | FL519006 | 0.112 | 0.151 | 0.888 | 1.637 |
| PDI like protein | Unclassified | CD051306 | 0.144 | 0.029 | 0.799 | 1.05 |
| Phi-1 | Unclassified | FL512435 | 0.41 | 0.705 | 0.934 | 1.057 |
| Transposase of Tn10 | Unclassified | FL512464 | 0.1 | 0.433 | 0.789 | 1.475 |
| **Cluster 2** | | | | | | |
| Polygalacturonase like protein | Cell defense | FL518998 | 0.621 | 1.239 | -1.816 | 1.221 |
| HSP | Cellular Organisation | FL518986 | 0.32 | 1.281 | -0.86 | 1.243 |
| Put SAP | Cellular Organisation | FL518999 | 1.15 | 1.124 | -0.834 | 0.956 |
| Put vacuolor ATP synthase | Energy metabolism | FL518930 | 0.734 | -0.105 | -0.667 | 0.642 |
| SAM synthetase | Hormone biosynthesis | FL518971 | 0.313 | 0.888 | -0.865 | 0.486 |
| Cyt P450 | Metabolism | FL518968 | 1.265 | 0.098 | -1.056 | 0.745 |
| Asparate aminotransferase | Metabolism | FL518970 | 0.512 | 0.401 | -0.591 | 1.223 |
| Mannose – 6 – Phos isomerase | Metabolism | FL518974 | 1.012 | 0.934 | -0.873 | 1.048 |
| P-protein like protein | Metabolism | FL518978 | 0.575 | 1.007 | -1.05 | 1.81 |
| Rubisco activase (small isoform) | Metabolism | FL518985 | 1.146 | 1.566 | -1.458 | 1.682 |
| Senescence | Protein degradation | FL518988 | 1.084 | 0.832 | -1.498 | 0.85 |
| Integral membrane protein | Signal transduction | FL518989 | 0.379 | 1.251 | -0.464 | 1.226 |
| Elongation factor 2 | Translation | FL512400 | 0.373 | 1.59 | -0.044 | 0.887 |
| Ribosomal protein L11 | Translation | FL518964 | 0.816 | 0.748 | -0.484 | 2.108 |
| Put ribosomal protein | Translation | FL518973 | 0.163 | 1.014 | -1.006 | 1.193 |
| 60s ribosomal protein L13a | Translation | FL518981 | 0.399 | 0.614 | -1.175 | 1.042 |
| Armadillo b catenin repeat | Unclassified | FL518965 | 0.306 | 1.214 | -1.012 | 0.972 |
| Predicted protein (cicer) | Unclassified | FL518969 | 0.179 | 1.208 | -0.613 | 0.377 |
| Hypo protein | Unclassified | FL518993 | 0.339 | 0.782 | -1.204 | 0.771 |
| **Cluster3** | | | | | | |
| Put chloroplast FtsH proteinase | Cell defense | FL518918 | 0.605 | 0.292 | 1.55 | 1.07 |
| Lipid transfer protein precursor | Cell defense | CD051321 | 0.034 | -0.377 | 1.637 | 1.098 |
| Aquaporin | Cell transport | FL512407 | 0.605 | -0.234 | 1.888 | 0.509 |
| Kinesin like protein | Cell transport | CD051276 | 0.151 | -0.599 | 1.782 | 1.345 |
| Put. Imbibition protein | Cellular Organisation | FL512365 | 1.079 | -0.494 | 0.516 | 0.851 |
| α –Tubulin | Cellular Organisation | FL512378 | 0.383 | 0.227 | 1.516 | 0.403 |
| Salt tolerant protein | Cellular Organisation | FL512396 | 1.078 | -0.599 | 1.782 | 0.627 |
| Integral mem. Protein | Cellular Organisation | FL512408 | 0.251 | 0.151 | 2.342 | 0.315 |
| Metallothionein | Cellular Organisation | FL512409 | 0.321 | -0.377 | 1.832 | 0.465 |
| SAP | Cellular Organisation | FL512411 | 0.895 | -0.667 | 1.683 | 0.239 |
| HSP | Cellular Organisation | FL518941 | 0.596 | 0.138 | 1.022 | 1.16 |
| Superoxide dismutase | Energy metabolism | FL512362 | 1.104 | 0.31 | 1.214 | 0.18 |
| Cationic peroxidase | Energy metabolism | FL512384 | 1.08 | -0.494 | 1.084 | 0.695 |
| Monooxygenase | Energy metabolism | FL512410 | 1.546 | -0.234 | 1.604 | 0.241 |
| Unsp. Monooxygenase | Energy metabolism | FL512427 | 0.649 | -0.286 | 0.731 | 0.328 |
| Lipoxygenase | Hormone biosynthesis | FL512369 | 0.189 | -0.12 | 0.669 | 0.619 |
| Allantoinase | Metabolism | FL512353 | 1.102 | 0.251 | 1.683 | 0.287 |
| Asn synthetase | Metabolism | FL512372 | 1.105 | -0.089 | 1.202 | 1.269 |
| Chlorophyll. a/b bp | Metabolism | FL512373 | 0.411 | -0.044 | 1.949 | 0.403 |
| Rubisco | Metabolism | FL512377 | 0.697 | -0.713 | 0.848 | 0.515 |
| Sucrose synthase | Metabolism | FL512424 | 0.293 | -0.415 | 0.895 | 0.777 |
| 1-L-myoinositol-1-P-synthetase | Metabolism | CD051303 | 0.282 | 0.07 | 1.269 | 0.283 |
| Enolase, isoform 1 | Metabolism | FL518916 | 0.835 | -0.103 | 0.81 | 1.357 |
| 1-deoxy-o-xylulose-5-Phos reductoisomerase | Metabolism | FL518921 | 0.707 | -0.411 | 1.158 | 1.218 |
| Glyceraldehydes-3-Phos dehydrogenase | Metabolism | FL518927 | 0.322 | 0.322 | 1.112 | 0.446 |
| α - 1,4-glucan phosphorylase | Metabolism | FL518943 | 0.605 | 0.111 | 2.815 | 0.64 |
| P-protein | Metabolism | FL518944 | 1.009 | 0.287 | 1.144 | 1.096 |
| Put apyrase | Metabolism | CD051304 | 0.832 | 0.014 | 1.084 | 1.214 |
| Trans-cinnamate-4-monooxygenase | Metabolism | CD051342 | 0.3 | -0.396 | 0.978 | -0.004 |
| Delta-8-sphingolipid desaturase | Metabolism | CD051350 | 0.282 | 0.07 | 1.269 | 0.283 |
| Long chain fatty acid condensing enzyme | Metabolism | CD051352 | 0.122 | -0.136 | 0.748 | -0.226 |
| Trehalose-6-phos 4H | Metabolism | CD051305 | 0.35 | -0.044 | 0.824 | 0.438 |
| UDP galactose-4-epimerase | Metabolism | FL512443 | 0.832 | 0.014 | 1.084 | 1.214 |
| Fructose-1,6-bisphosphatase | Metabolism | FL512453 | 0.731 | -0.136 | 0.807 | 0.745 |
| Asparagines synthetase | Metabolism | FL512455 | 0.918 | -0.201 | 0.799 | 0.661 |
| Polyubiquitin | Protein degradation | FL512426 | 0.3 | -0.396 | 0.978 | -0.004 |
| Ca bp | Signal Transduction | FL512355 | 0.309 | -0.737 | 1.124 | 0.759 |
| Stress related protein | Signal Transduction | FL512361 | 0.433 | -0.761 | 1.144 | 0.873 |
| Phosphoglycerate kinase | Signal Transduction | FL512374 | 1.41 | -0.136 | 1.459 | 0.745 |
| Protein kinase family(arabi) | Signal Transduction | FL512375 | 1.11 | -0.201 | 0.799 | 0.661 |
| Receptor like protein kinase | Signal Transduction | FL512376 | 0.41 | -0.667 | 0.934 | 0.238 |
| Put Zn bp | Signal Transduction | FL512395 | 1.145 | -0.494 | 1.687 | 0.144 |
| IAA-AA hydrolase | Signal Transduction | FL512418 | 1.134 | -0.304 | 1.982 | 0.263 |
| CBL-interacting protein kinase | Signal Transduction | FL512472 | 0.208 | 0.263 | 1.551 | 0.02 |
| Omnipotent suppressor protein | Signal Transduction | FL519009 | 0.208 | 0.263 | 1.551 | 0.02 |
| Transcription factor BTF3 | Transcription | FL512406 | 0.135 | 0.202 | 1.275 | 0.224 |
| Transcription factor Myb-1 | Transcription | FL512419 | 0.034 | -0.377 | 2.154 | 0.235 |
| 60S ribosomal protein L37a | Translation | FL512367 | 0.963 | -0.434 | 1.007 | 0.34 |
| Elongation factor 2 | Translation | FL512382 | 0.556 | -0.286 | 0.895 | 1.07 |
| Ribosomal protein L18a | Translation | FL518931 | 0.275 | -0.029 | 3.479 | 0.729 |
| 40S ribosomal protein S7 homolog | Translation | CD051338 | 0.293 | -0.415 | 0.895 | 0.777 |
| SOUL protein | Unclassified | FL518983 | 0.489 | 0.07 | 0.84 | 0.934 |
| PPF-1 protein | Unclassified | CD051344 | 0.649 | -0.286 | 0.731 | 0.328 |
| Unknown protein (Arabi) | Unclassified | FL512371 | 1.081 | -0.556 | 1.257 | 1.292 |
| Cold induced alfalfa gene | Unclassified | FL512473 | 0.122 | -0.136 | 0.748 | -0.226 |
| Put protein | Unclassified | CD051275 | 1.132 | 0.151 | 1.05 | 0.315 |
| **Cluster4** | | | | | | |
| 40S ribosomal protein S25 | Translation | FL518942 | 0.36 | -3.644 | 1.021 | 0.363 |
| **Cluster5** | | | | | | |
| Ribosomal protein L3 | Translation | FL518934 | 0.433 | -3.837 | -0.209 | 1.808 |
| **Cluster6** | | | | | | |
| Put ER lumen protein retaining receptor | Cellular Organisation | FL518946 | 0.625 | -5.644 | 0.92 | 1.962 |
| **Cluster7** | | | | | | |
| Ceramidase family protein | Cellular Organisation | FL518915 | 0.605 | -2.238 | -1.162 | 1.01 |
| **Cluster8** | | | | | | |
| FtsH like protein PftF precursor | Cell defense | FL518951 | 0.1 | -1.515 | 0.789 | 1.953 |
| Prolyl-4-hydroxylase | Cellular Organisation | CD051295 | 0.275 | -0.916 | 0.766 | 1.804 |
| Nucleolar protein | Cellular Organisation | FL512465 | 0.614 | -1.434 | 1.503 | 1.158 |
| GDP mannose pyrophosphorylase | Metabolism | FL512412 | 0.287 | -0.916 | 0.766 | 1.804 |
| 1-acyl-sn-glycerol-3-Phos acyltransferase | Metabolism | FL518917 | 0.374 | -1.283 | 0.679 | 1.202 |
| Pyruvate dehydrogenase E1 b | Metabolism | FL518950 | 0.44 | -1.286 | 0.547 | 1.733 |
| Photolyase | Signal transduction | FL518952 | 0.614 | -1.434 | 1.503 | 1.158 |
| **Cluster9** | | | | | | |
| Basic blue Cu protein | Cell defense | FL512383 | 0.275 | -0.971 | 0.651 | 0.714 |
| HSP70 | Cellular Organisation | FL512415 | 0.229 | -0.578 | 0.322 | 0.708 |
| Probable HSP | Cellular Organisation | FL518913 | 1.642 | -0.941 | -0.407 | 1.643 |
| Vacuolor ATP synthetase | Energy metabolism | FL512392 | 0.724 | -0.667 | 0.098 | 0.732 |
| Phosphate translocator | Signal Transduction | FL512425 | 1.239 | -1.322 | 0.124 | 1.005 |
| Put. RT | Transcription | FL512380 | 0.16 | -0.667 | 0.333 | 1.256 |
| UV opsin | Unclassified | FL512363 | 1.079 | -0.69 | 0.623 | 0.617 |
| Putative protein | Unclassified | CD051298 | 0.281 | -0.713 | 0.411 | 0.439 |
| Albumin 2 | Unclassified | FL512461 | 0.724 | -0.667 | 0.098 | 0.732 |
| **Cluster10** | | | | | | |
| Lipid transfer protein | Cell defense | FL512385 | 0.212 | 1.043 | 1.379 | 1.228 |
| Lipid transfer protein | Cell defense | FL512385 | 0.379 | 1.299 | 1.195 | 1.692 |
| Class 10 PR protein | Cell defense | FL512394 | 1.024 | 1.379 | 0.622 | 1.958 |
| MRP1 | Cell defense | FL512449 | 0.163 | 1.064 | 1 | 1.134 |
| Elicitor inducible gene | Cell defense | FL512456 | 0.484 | 0.642 | 1.104 | 1.39 |
| Aquaporin like Water channel protein | Cell transport | FL512354 | 0.135 | 1.05 | 2.281 | 2.106 |
| Dehydrin | Cellular Organisation | FL512347 | 0.715 | 1.316 | 1.091 | 1.085 |
| Chalcone synthetase | Cellular Organisation | FL512402 | -0.112 | 1 | 0.888 | 2.18 |
| RNA bp | Cellular Organisation | FL518922 | 0.11 | 0.614 | 1.277 | 2.741 |
| Salt tolerance protein 4 | Cellular Organisation | FL518936 | 0.692 | 1.322 | 1.584 | 1.154 |
| Cellulase synthase | Cellular Organisation | FL518949 | 1.235 | 0.731 | 0.85 | 1.683 |
| Adenine ntd translocator | Cellular Organisation | FL518967 | 0.299 | 0.782 | 0.7 | 1.764 |
| HSP | Cellular Organisation | FL518996 | 1.064 | 2.141 | 0.308 | 1.79 |
| RNA bp | Cellular Organisation | FL512337 | 0.595 | 0.986 | 1.521 | 1.59 |
| Metallothionein | Cellular Organisation | FL512338 | 0.978 | 1.151 | 1.683 | 1.77 |
| Glycine rich protein | Cellular Organisation | FL512344 | 0.354 | 1.642 | 1.526 | 1.566 |
| Imbibition protein | Cellular Organisation | FL519000 | 1.079 | 1.151 | 1.475 | 1.782 |
| Seed maturation protein | Cellular Organisation | FL519001 | 0.411 | 0.526 | 1.516 | 1.731 |
| Histone 2A | Cellular Organisation | CD051290 | 0.214 | 0.903 | 0.956 | 1.124 |
| Dehydrin | Cellular Organisation | CD051297 | 1.322 | 1.22 | 1.428 | 1.828 |
| LEA protein 2 | Cellular Organisation | CD051326 | 1.106 | 1.379 | 1.58 | 1.642 |
| LEA-1 | Cellular Organisation | CD051271 | 1.157 | 1.104 | 1.275 | 1.674 |
| Dehydrin1 | Cellular Organisation | FL512434 | 0.669 | 1.057 | 1.556 | 1.669 |
| Triose Phos translocator | Cellular Organisation | FL512441 | 0.74 | 0.864 | 1.029 | 1.516 |
| Annexin | Cellular Organisation | FL512442 | 0.275 | 0.595 | 1.077 | 1.406 |
| Probable K+ transporter | Cellular Organisation | FL512444 | 0.212 | 1.077 | 1.379 | 1.228 |
| Aconitase family | Cellular Organisation | FL512448 | 0.356 | 1.384 | 0.84 | 1.281 |
| Dehydration induced protein | Cellular Organisation | FL512471 | 0.74 | 1.77 | 1.996 | 2.266 |
| Cu/Zn superoxide dismutase II | Energy metabolism | FL512366 | 0.428 | 1.293 | 1.618 | 1.634 |
| Catalase fragment | Energy metabolism | FL518979 | 0.287 | 1.131 | 1.454 | 1.2 |
| Unspecific monooxygenase | Energy metabolism | FL518994 | 0.744 | 2.101 | 0.379 | 3.569 |
| P type H+ATPase | Energy metabolism | CD051280 | 1.47 | 1.59 | 1.872 | 1.58 |
| Thioredoxin F type, chloroplast precursor | Energy metabolism | CD051307 | 0.516 | 1.077 | 1.356 | 1.48 |
| Mitochondrial uncoupling protein | Energy metabolism | CD051283 | 0.993 | 0.888 | 1.585 | 1.124 |
| Methionine adenosyl transferase | Hormone biosynthesis | FL518953 | 0.316 | 0.595 | 1.535 | 1.984 |
| SAM | Hormone biosynthesis | CD051262 | 0.401 | 0.642 | 1.333 | 1.214 |
| Lipoxygenase | Hormone biosynthesis | CD051273 | 0.642 | 1.245 | 0.748 | 1.201 |
| Lipoxygenase | Hormone biosynthesis | CD051273 | 0.605 | 0.895 | 1.888 | 1.281 |
| Rubisco activase | Metabolism | FL512364 | 0.275 | 0.971 | 0.856 | 1.314 |
| Light harvesting protein | Metabolism | FL518912 | 0.623 | 1.146 | 1.742 | 2.144 |
| AMP bp | Metabolism | FL518923 | 0.503 | -0.013 | 1.299 | 1.968 |
| Caffeic acid-o-methyltransferase | Metabolism | FL518929 | 0.27 | 0.189 | 0.806 | 2.121 |
| Rubisco activase b form precursor | Metabolism | FL518947 | 0.163 | 0.098 | 1.327 | 1.997 |
| Carbonic anhydrase | Metabolism | FL518948 | 0.231 | 1.485 | 1.847 | 2.104 |
| Put C type cyt synthase | Metabolism | FL518957 | 0.498 | 1.138 | 0.859 | 1.809 |
| Rubisco activase | Metabolism | FL518984 | 0.422 | 0.401 | 1.007 | 1.559 |
| w-6-desaturase | Metabolism | FL518987 | 0.43 | 0.978 | 2.762 | 2.407 |
| Hydroxyacyl glutathione hydrolase | Metabolism | FL518990 | 0.888 | 1.043 | 0.597 | 1.206 |
| Glycolate oxidase | Metabolism | FL512340 | 1.163 | 0.978 | 1.189 | 1.58 |
| Cysteine synthase | Metabolism | FL512341 | 0.212 | 1.077 | 1.35 | 1.22 |
| Alcohol dehydrogenase | Metabolism | CD051265 | 1.079 | 0.949 | 1.157 | 1.339 |
| β-amylase | Metabolism | CD051266 | 0.614 | 1.417 | 1.687 | 2.284 |
| ADP glucose pyrophosphorylase | Metabolism | CD051279 | 0.963 | 1.05 | 1.287 | 1.345 |
| Phosphoribosylanthranilate transferase like protein | Metabolism | CD051285 | 0.428 | 1 | 1.281 | 1.357 |
| Cinnamoyl CoA reductase | Metabolism | CD051301 | 0.74 | 0.864 | 1.029 | 1.516 |
| Trehalose-6-Phos synthase homolog | Metabolism | CD051305 | 0.212 | 1.077 | 1.379 | 1.228 |
| Aldehyde dehydrogenase family | Metabolism | FL519005 | 0.163 | 1.064 | 1 | 1.134 |
| Phosphoglucomutase | Metabolism | CD051347 | 0.373 | 1.411 | 1.575 | 1.585 |
| Put β-amylase | Metabolism | FL519010 | 1.057 | 1.411 | 1.58 | 1.609 |
| Put chloroplast targeted b amylase | Metabolism | FL512429 | 0.428 | 1 | 1.281 | 1.357 |
| Lipase(class 3)family | Metabolism | FL512437 | 0.383 | 0.345 | 1.516 | 1.748 |
| Put alkaline neutral invertase | Metabolism | FL512451 | 0.724 | 0.807 | 1.036 | 1.58 |
| Thioprotease | Protein degradation | FL512333 | 0.888 | 1.157 | 1.59 | 1.57 |
| Early leaf senescence abundant cysteine proteinase | Protein degradation | FL512339 | 1.22 | 1.05 | 2.281 | 2.106 |
| Cysteine proteinase | Protein degradation | FL512342 | 0.411 | 2.316 | 1.47 | 1.531 |
| Cysteine proteinase type protein | Protein degradation | CD051336 | 1.214 | 1.293 | 1.848 | 1.683 |
| ATP dependent clp protease | Protein degradation | CD051341 | 1.131 | 1.144 | 1.868 | 1.195 |
| Ubiquitin conjugating protein | Protein degradation | CD051293 | 0.918 | 1.163 | 1.511 | 1.651 |
| Put ubiquitin | Protein degradation | FL512431 | 0.731 | 0.807 | 1.202 | 1.345 |
| 14-3-3 brain protein homolog | Signal Transduction | FL512351 | 1.011 | 0.401 | 2.151 | 1.16 |
| Nodule enhanced sucrose synthase | Signal Transduction | FL518926 | 1.9 | 1.144 | 0.547 | 1.09 |
| α-NAC | Signal Transduction | FL518992 | 0.484 | 1.31 | 0.048 | 2.777 |
| Put nucleolar GTP bp | Signal Transduction | FL512332 | 0.516 | 1.202 | 1.263 | 1.379 |
| WD repeat protein like protein | Signal Transduction | CD051264 | 1.235 | 0.993 | 1.144 | 1.275 |
| Put protein kinase | Signal Transduction | CD051343 | 1.233 | 1.293 | 1.618 | 1.687 |
| Nodule enhanced protein phosphatase | Signal Transduction | FL519004 | 0.356 | 1.384 | 0.84 | 1.281 |
| G-protein coupled receptor like protein | Signal Transduction | CD051322 | 1.079 | 1.526 | 1.475 | 1.664 |
| ADP ribosylation factor like protein | Signal Transduction | CD051324 | 0.724 | 0.705 | 1.036 | 1.58 |
| Jasmonic acid 2 | Signal Transduction | CD051357 | 1.111 | 1.669 | 1.687 | 1.811 |
| Protein kinase | Signal Transduction | CD051317 | 1.134 | 0.993 | 1.687 | 1.189 |
| SOS2 like protein | Signal Transduction | FL512440 | 0.642 | 1.245 | 1.422 | 1.722 |
| Put mem protein | Signal Transduction | FL512450 | 1.079 | 1.526 | 1.475 | 1.664 |
| High affinity Fe+2-Pb+2 permease | Signal Transduction | FL512457 | 0.722 | 0.731 | 0.85 | 1.683 |
| Protein phosphatase 2C | Signal Transduction | CD051312 | 1.07 | 1.043 | 1.876 | 1.228 |
| Glycerol kinase related | Signal Transduction | FL512459 | 0.299 | 0.333 | 1.293 | 1.824 |
| Zn finger protein | Transcription | FL512348 | 0.926 | 1.091 | 1.57 | 1.421 |
| RNA helicase | Transcription | CD051282 | 0.411 | 1.233 | 1.47 | 1.59 |
| Put Zn finger protein | Transcription | CD051330 | 0.632 | 0.623 | 1.35 | 1.876 |
| AP2 domain like protein | Transcription | CF074502 | 1.077 | 1.35 | 1.406 | 1.637 |
| Transcriptional repressor of GlcNag | Transcription | FL519012 | 1.177 | 1.281 | 2.293 | 1.57 |
| Zn finger protein | Transcription | FL512439 | 0.642 | 1.22 | 1.884 | 1.828 |
| Dehydration responsive element bp3 | Transcription | FL512463 | 0.731 | 1.281 | 1.299 | 1.733 |
| DEAD box RNA helicase | Translation | FL512356 | 0.212 | 1.077 | 1.35 | 1.147 |
| Translation initiation factor 5A | Translation | FL512389 | 0.356 | 1.384 | 0.84 | 1.179 |
| Ribosomal protein L17 | Translation | FL518938 | 0.332 | 0.526 | 0.767 | 1.972 |
| 40S ribosomal protein S9 like | Translation | FL518963 | 0.484 | 0.642 | 1.104 | 1.39 |
| 40S ribosomal protein;P6-1 | Translation | FL518982 | 0.662 | 0.941 | 0.952 | 1.535 |
| 40S ribosomal protein S13 | Translation | FL512445 | 0.516 | 1.077 | 1.356 | 1.48 |
| 60S ribosomal protein L27A | Translation | FL512452 | 0.202 | 1.379 | 1.58 | 1.642 |
| 60S ribosomal protein L37A | Translation | CD051286 | 0.623 | 0.748 | 1.257 | 1.292 |
| Eukaryotic translation initiation factor 5A-1 | Translation | CD051287 | 0.731 | 0.536 | 1.202 | 1.345 |
| 40S ribosomal protein S11 | Translation | CD051346 | 0.506 | 0.986 | 1.214 | 1.47 |
| Ribosomal protein S15 | Translation | CD051284 | 0.895 | 0.705 | 1.144 | 1.31 |
| PB-1 domain containing protein | Unclassified | FL518925 | 1.115 | 0.705 | 0.612 | 1.41 |
| Callus protein P23 | Unclassified | FL518939 | 0.625 | 0.696 | 1.899 | 1.569 |
| Put KH domain protein | Unclassified | FL518977 | 0.394 | 1.384 | 1.212 | 1.166 |
| Put. Protein (Arabi.) | Unclassified | FL512370 | 0.428 | 1 | 1.501 | 1.357 |
| Put. Leunig/B-120 | Unclassified | FL512477 | 1.177 | 1.281 | 2.293 | 1.57 |
| Phi – 1 like protein | Unclassified | CD051263 | 0.722 | 0.31 | 1.214 | 1.411 |
| Cold induced alfalfa gene | Unclassified | CD051294 | 0.383 | 0.345 | 1.516 | 1.748 |
| Put protein | Unclassified | FL519003 | 1.145 | 0.595 | 1.077 | 1.406 |
| Hyp protein | Unclassified | CD051269 | 0.244 | 1.22 | 1.157 | 1.417 |
| Profiling | Unclassified | FL512430 | 0.623 | 0.748 | 1.257 | 1.292 |
| VTC2 | Unclassified | FL512432 | 0.411 | 0.526 | 1.516 | 1.731 |
| Put cullin | Unclassified | FL512458 | 0.379 | 1.299 | 1.195 | 1.692 |
| Zwille protein | Unclassified | FL512467 | 0.316 | 0.595 | 1.535 | 1.984 |
| **Cluster11** | | | | | | |
| MRP like ABC transporter | Cell defense | FL512349 | 1.147 | 1.057 | 2.242 | 0.465 |
| Disease resistance response protein | Cell defense | FL512398 | 1.07 | 1.144 | 1.918 | 0.299 |
| MRP like ABC transporter | Cell defense | FL518997 | 1.049 | 1.189 | 2.371 | 0.862 |
| Polygalacturonase inhibiting protein | Cell defense | CD051270 | 0.993 | 1.521 | 1.48 | 0.632 |
| Non specific lipid transfer | Cell defense | FL512469 | 1.038 | 1.74 | 1.899 | 0.352 |
| Kinesin like protein | Cell transport | CD051276 | 0.275 | 1.47 | 1.832 | 0.465 |
| Proline rich protein | Cellular Organisation | FL512352 | 0.197 | 1.475 | 1.856 | 0.643 |
| Put. RNA bp | Cellular Organisation | FL512359 | 0.354 | 1.642 | 1.575 | 0.328 |
| Seed protein | Cellular Organisation | FL512360 | 0.507 | 0.642 | 1.333 | 0.225 |
| RNA bp cp29 protein | Cellular Organisation | FL512387 | 0.261 | 1.111 | 1.531 | 0.37 |
| Put. Proline rich protein APG | Cellular Organisation | FL512405 | 0.244 | 1.761 | 0.585 | 0.31 |
| Importin | Cellular Organisation | FL512417 | 1.227 | 1.098 | 1.287 | 0.154 |
| Ripening induced protein | Cellular Organisation | FL512428 | 0.197 | 1.163 | 0.766 | 0.33 |
| α - tubuline | Cellular Organisation | FL518958 | 0.463 | 1.214 | 0.919 | 0.358 |
| HVA22 homolog | Cellular Organisation | CD051272 | 0.209 | 1.111 | 1.491 | 0.521 |
| Nitrate transporter | Cellular Organisation | FL512438 | 0.993 | 1.521 | 1.48 | 0.632 |
| Endomembrane protein 70 | Cellular Organisation | FL512454 | 0.623 | 0.714 | 1.036 | 0.755 |
| Cyt. P450monooxygenase | Energy metabolism | FL518911 | 0.918 | 1.163 | 0.888 | 0.188 |
| Cu/Zn superoxide dismutase II | Energy metabolism | FL512366 | 0.373 | 0.872 | 0.809 | 0.314 |
| Cyt. P450monooxygenase | Energy metabolism | FL518911 | 0.963 | 0.696 | 1.499 | 0.684 |
| Cyt P450 dependent subunit | Energy metabolism | CD051325 | 0.222 | 1.245 | 2.098 | 0.308 |
| Put-3-isopropylmalate dehydrogenase | Energy metabolism | FL512446 | 0.261 | 1.111 | 1.531 | 0.37 |
| Fructose biphos. Aldose | Metabolism | FL512350 | 0.31 | 1.05 | 0.971 | -0.076 |
| Chloroplast translocon | Metabolism | FL512420 | 0.222 | 1.245 | 2.098 | 0.308 |
| β – cobalamine synthase | Metabolism | FL512422 | 0.19 | 0.575 | 1.091 | 0.457 |
| Met. synthase | Metabolism | FL512423 | 0.286 | 1.546 | 1.157 | 0.649 |
| Leuco-anthocynidine dioxygenase | Metabolism | CD051278 | 0.209 | 1.111 | 1.491 | 0.521 |
| Ketol acid reductoisomerase | Metabolism | FL518933 | 0.197 | 1.696 | 1.146 | 0.604 |
| Methionin synthase | Metabolism | FL518940 | 0.643 | 1.07 | 2.535 | 0.437 |
| Nucleotide sugar epimerase like protein | Metabolism | FL518945 | 1.007 | 1.585 | 1.331 | 0.467 |
| Put amino peptidase | Metabolism | FL518960 | 0.394 | 1.47 | 1.542 | 0.63 |
| Malate dehydrogenase | Metabolism | FL518972 | 1.276 | 0.848 | 0.68 | 0.731 |
| Rubisco | Metabolism | FL518995 | 0.165 | 3.136 | 1.163 | 0.677 |
| Rubisco activase(small isoform) | Metabolism | CD051311 | 0.35 | 1.021 | 0.848 | 0.401 |
| Phophonopyruvate decarboxylase like protein | Metabolism | FL519008 | 0.19 | 0.575 | 1.091 | 0.457 |
| Raffinose synthase | Metabolism | FL512447 | 0.35 | 1.021 | 0.848 | 0.401 |
| Glycosyl hydrolase family 14 | Metabolism | FL512460 | 0.322 | 1.007 | 1.124 | 0.476 |
| Prolyl-peptidyl isomerase | Protein degradation | FL512388 | 0.35 | 1.021 | 0.848 | 0.27 |
| Monoubiquitin | Protein degradation | FL518932 | 0.219 | 1.757 | 1.295 | 0.46 |
| Cysteine proteinase type protein | Protein degradation | CD051336 | 0.399 | 1.202 | 1.322 | 1.042 |
| Ubiquitin homolog | Protein degradation | FL512466 | 0.354 | 1.642 | 1.575 | 0.328 |
| ADP ribosylation factor like protein | Signal Transduction | FL512368 | 0.322 | 1.007 | 1.124 | 0.476 |
| Put WD40 repeat protein | Signal Transduction | FL512386 | 1.271 | 1.077 | 1.356 | 0.743 |
| Put ABA responsive protein | Signal Transduction | FL512397 | 1.214 | 1.104 | 1.848 | 0.281 |
| Membrane protein/B-12 | Signal Transduction | FL512474 | 0.114 | 0.757 | 1.138 | 0.429 |
| Protein kinase family protein | Signal transduction | FL512375 | 1.346 | 0.614 | 1.036 | 0.755 |
| Nodule enhanced phophorus protein | Signal Transduction | FL519011 | 0.111 | 0.546 | 1.428 | 0.396 |
| RAP2.6 | Transcription | CD051355 | 0.261 | 1.111 | 1.531 | 0.37 |
| Put AP2 domain transcriptional regulator | Transcription | FL519007 | 1.227 | 1.651 | 1.782 | 1.131 |
| Aspartyl-tRNA synthetase | Transcription | FL512433 | 1.41 | 1.541 | 1.459 | 0.745 |
| 40s ribosomal protein 57 homolog | Translation | FL512399 | 0.506 | 1.428 | 0.856 | 0.812 |
| Elongation factor 1 alpha | Translation | FL518919 | 1.644 | 2.325 | 4.373 | 0.333 |
| 60S ribosomal protein L3 | Translation | FL518937 | 0.402 | 1 | 1.16 | 0.017 |
| Ribosome associated protein p40 | Translation | FL518954 | 0.07 | 1.74 | 2.95 | 0.352 |
| Ribosomal protein S14 | Translation | FL518961 | 0.283 | 1.111 | 1.194 | 0.729 |
| Ribosomal protein S2 | Translation | CD051333 | 0.286 | 1.546 | 1.157 | 0.649 |
| Unknown protein | Unclassified | FL518975 | 0.942 | 1.333 | 2.121 | 0.377 |
| PPF-1 protein | Unclassified | CD051334 | 0.197 | 1.163 | 0.766 | 0.33 |
| Leu rich recepter like protein | Unclassified | FL512357 | 0.009 | 2.316 | 0.714 | 0.403 |
| Put CCR-4 associated factor | Unclassified | FL512393 | 1.106 | 1.379 | 0.799 | 0.649 |
| Zwille protein shoot meristem | Unclassified | FL512476 | 0.111 | 0.546 | 1.428 | 0.396 |
| Serine rich protein | Unclassified | CD051340 | 0.24 | 1.111 | 0.903 | 0.202 |
| Drm3 | Unclassified | CD051331 | 0.275 | 0.774 | 1.687 | 0.144 |
