# Supplementary material for: Analysis of gene expression in response to water deficit of chickpea (Cicer arietinum L.) varieties differing in drought tolerance
Source: BMC Plant Biol. 2010 Feb 9;10:24. doi: 10.1186/1471-2229-10-24 (PMC2831037; doi:10.1186/1471-2229-10-24)
Supplement: Additional file 4 — Transcript expression profiles of selected 53 genes in PUSABGD72 seedlings. Transcript expression analysis in response to drought stress at different time points with the fold-expression values. [Standard deviations (SD ±) are calculated from three different experiments. The transcripts are listed according to their putative functions]. [file 1471-2229-10-24-S4.DOC]

## Additional File 4 - Transcript expression profiles of selected 53 genes in PUSABGD72 seedlings

Transcript expression analysis in response to drought stress at different time points with the fold-expression values. [Standard deviations (SD±) are calculated from three different experiments. The transcripts are listed according to their putative function].

| **EST Accession No.** | **E-value** | **Putative fuction** | **C** | **SD(±)** | **3d** | **SD(±)** | **6d** | **SD(±)** | **12d** | **SD(±)** |
| --- | --- | --- | --- | --- | --- | --- | --- | --- | --- | --- |
| **Cell Defense** | | | |  |  |  |  |  |  |  |
| FL512398 | 1.00E-43 | Disease resistance response protein | 2.10 | 0.09 | 2.21 | 0.14 | 3.78 | 0.16 | 1.23 | 0.10 |
| FL512394 | 1.00E-23 | Class 10 PR protein | 2.03 | 0.20 | 2.60 | 0.27 | 1.54 | 0.16 | 3.88 | 0.23 |
| FL512357 | 4.00E-05 | Leu rich recepter like protein | 1.01 | 0.22 | 4.98 | 0.19 | 1.64 | 0.13 | 1.32 | 0.15 |
| **Cell transport** | | |  |  |  |  |  |  |  |  |
| FL512354 | 4.00E-51 | Aquaporin like Water channel protein | 2.10 | 0.20 | 2.07 | 0.11 | 0.12 | 0.12 | 4.30 | 0.18 |
| FL512349 | 8.00E-77 | MRP like ABC transporter | 2.21 | 0.07 | 2.08 | 0.15 | 4.73 | 0.22 | 1.38 | 0.11 |
| FL518997 | 7.00E-43 | MRP like ABC transporter | 2.07 | 0.21 | 2.28 | 0.14 | 5.17 | 0.04 | 1.82 | 0.16 |
| **Cellular Organisation** | | |  |  |  |  |  |  |  |  |
| FL512450 | 1.00E-20 | Put mem protein | 2.11 | 0.12 | 2.88 | 0.14 | 2.78 | 0.14 | 3.17 | 0.14 |
| FL512469 | 9.00E-14 | Non specific lipid transfer | 2.05 | 0.09 | 3.34 | 0.14 | 3.73 | 0.11 | 1.28 | 0.06 |
| FL512352 | 3.00E-15 | Proline rich protein | 2.15 | 0.27 | 2.78 | 0.10 | 3.62 | 0.18 | 1.56 | 0.29 |
| FL512405 | 4.00E-79 | Put. Proline rich protein | 2.18 | 0.01 | 3.39 | 0.13 | 1.50 | 0.14 | 1.24 | 0.01 |
| FL518949 | 1.00E-10 | Cellulase synthase | 2.35 | 0.34 | 1.66 | 0.16 | 1.80 | 0.14 | 3.21 | 0.17 |
| FL518996 | 6.00E-77 | HSP 70 cognate | 2.09 | 0.08 | 4.41 | 0.10 | 1.24 | 0.14 | 3.46 | 0.21 |
| FL519000 | 2.00E-38 | Imbibition protein | 2.11 | 0.22 | 2.22 | 0.21 | 2.78 | 0.17 | 3.44 | 0.15 |
| CD051297 | 3.00E-25 | Dehydrin | 2.50 | 0.29 | 2.33 | 0.15 | 2.69 | 0.22 | 3.55 | 0.20 |
| CD051326 | 6.00E-37 | LEA protein 2 | 2.15 | 0.15 | 2.60 | 0.14 | 2.99 | 0.04 | 3.12 | 0.13 |
| CD051271 | 6.00E-16 | LEA-1 | 2.23 | 0.05 | 2.15 | 0.13 | 2.42 | 0.16 | 3.19 | 0.18 |
| **Energy metabolism** | | |  |  |  |  |  |  |  |  |
| FL512338 | 2.00E-26 | Metallothionein | 2.04 | 0.17 | 2.22 | 0.13 | 3.21 | 0.18 | 3.41 | 0.14 |
| FL512366 | 8.00E-62 | Cu/Zn superoxide dismutase II | 2.15 | 0.17 | 2.45 | 0.19 | 3.07 | 0.14 | 3.10 | 0.16 |
| CD051280 | 1.00E-11 | P type H^+^ATPase | 2.77 | 0.27 | 3.01 | 0.07 | 3.66 | 0.14 | 2.99 | 0.10 |
| **Metabolism** |  |  |  |  |  |  |  |  |  |  |
| FL512353 | 4.00E-31 | Allantoinase | 2.15 | 0.10 | 1.19 | 0.11 | 3.21 | 0.03 | 1.22 | 0.11 |
| FL518945 | 2.00E-92 | Nucleotide sugar epimerase like protein | 2.01 | 0.12 | 3.00 | 0.18 | 2.52 | 0.12 | 1.38 | 0.12 |
| CD051266 | 1.00E-89 | β-amylase | 2.00 | 0.23 | 2.67 | 0.08 | 3.22 | 0.13 | 4.87 | 0.02 |
| FL519010 | 2.00E-90 | Put β-amylase | 2.08 | 0.24 | 2.66 | 0.13 | 2.99 | 0.29 | 3.05 | 0.16 |
| FL518926 | 4.00E-32 | Nodule enhanced sucrose synthase | 3.73 | 0.41 | 2.21 | 0.09 | 1.46 | 0.23 | 2.13 | 0.15 |
| **Protein degradation** | | |  |  |  |  |  |  |  |  |
| FL512339 | 1.00E-72 | Early leaf senescence abundant cysteine proteinase | 2.33 | 0.02 | 2.07 | 0.16 | 4.86 | 0.22 | 4.30 | 0.27 |
| CD051336 | 1.00E-52 | Cysteine proteinase type protein | 2.32 | 0.13 | 2.45 | 0.16 | 3.60 | 0.06 | 3.21 | 0.08 |
| CD051341 | 8.00E-73 | ATP dependent clp protease | 2.19 | 0.07 | 2.21 | 0.19 | 3.65 | 0.14 | 2.29 | 0.06 |
| CD051293 | 9.00E-56 | Ubiquitin conjugating protein | 2.19 | 0.06 | 2.24 | 0.17 | 2.85 | 0.14 | 3.14 | 0.28 |
| **Signal Transduction** | | |  |  |  |  |  |  |  |  |
| FL512351 | 3.00E-56 | 14-3-3 brain protein homolog | 2.02 | 0.06 | 1.32 | 0.24 | 4.44 | 0.21 | 2.24 | 0.07 |
| CD051343 | 3.00E-34 | Put protein kinase | 2.35 | 0.02 | 2.45 | 0.03 | 3.07 | 0.07 | 3.22 | 0.23 |
| CD051322 | 5.00E-63 | G-protein coupled receptor like protein | 2.11 | 0.15 | 2.88 | 0.18 | 2.78 | 0.06 | 3.17 | 0.06 |
| CD051357 | 2.00E-19 | Jasmonic acid 2 | 2.16 | 0.02 | 3.18 | 0.22 | 3.22 | 0.15 | 3.51 | 0.06 |
| CD051317 | 8.00E-05 | Protein kinase (CIPK25) | 2.19 | 0.06 | 1.99 | 0.14 | 3.22 | 0.16 | 2.28 | 0.19 |
| FL512440 | 1.00E-43 | SOS2 like protein (CIPK6) | 2.19 | 0.34 | 2.37 | 0.18 | 2.68 | 0.26 | 3.30 | 0.16 |
| CD051312 | 5.00E-48 | Protein phosphatase 2C | 2.10 | 0.19 | 2.06 | 0.19 | 3.67 | 0.14 | 2.34 | 0.15 |
| FL512472 | 6.00E-23 | CBL-interacting protein kinase | 1.16 | 0.15 | 1.20 | 0.14 | 2.93 | 0.03 | 1.01 | 0.14 |
| **Transcription** |  |  |  |  |  |  |  |  |  |  |
| CF074502 | 4.00E-17 | AP2 domain like protein | 2.11 | 0.07 | 2.55 | 0.09 | 2.65 | 0.11 | 3.11 | 0.07 |
| FL519007 | 9.00E-09 | Put AP2 domain transcriptional regulator | 2.34 | 0.04 | 3.14 | 0.23 | 3.44 | 0.17 | 2.19 | 0.07 |
| FL519012 | 2.00E-46 | Transcriptional repressor of GlcNag | 2.26 | 0.02 | 2.43 | 0.12 | 4.90 | 0.12 | 2.97 | 0.07 |
| FL512439 | 5.00E-16 | Zn finger protein | 2.19 | 0.21 | 2.33 | 0.13 | 3.69 | 0.12 | 3.55 | 0.13 |
| FL512463 | 2.00E-19 | Dehydration responsive element bp3 | 2.14 | 0.23 | 2.43 | 0.13 | 2.46 | 0.51 | 3.32 | 0.18 |
| FL518992 | 2.00E-09 | α-NAC | 2.14 | 0.26 | 2.48 | 0.19 | 1.03 | 0.20 | 6.86 | 0.17 |
| FL512359 | 5.00E-23 | Put. RNA bp | 2.28 | 0.04 | 3.12 | 0.16 | 2.98 | 0.18 | 1.26 | 0.08 |
| **Translation** |  |  |  |  |  |  |  |  |  |  |
| FL518919 | 1.00E-52 | Elongation factor 1 alpha | 3.13 | 0.24 | 5.01 | 0.16 | 20.72 | 0.18 | 1.26 | 0.17 |
| FL512452 | 6.00E-19 | 60S ribosomal protein L27A | 2.15 | 0.23 | 2.60 | 0.21 | 2.99 | 0.18 | 3.12 | 0.15 |
| FL518931 | 2.00E-34 | Ribosomal protein L18a | 1.21 | 0.13 | 0.98 | 0.12 | 11.15 | 0.18 | 1.66 | 0.15 |
| FL518954 | 4.00E-83 | Ribosome associated protein p40 | 1.05 | 0.17 | 3.34 | 0.16 | 7.73 | 0.21 | 1.28 | 0.18 |
| **Unclassified** |  |  |  |  |  |  |  |  |  |  |
| FL512477 | 4.00E-17 | Put. Leunig | 2.26 | 0.25 | 2.43 | 0.03 | 4.90 | 0.17 | 2.97 | 0.25 |
| FL512471 | 6.00E-28 | Dehydration induced protein | 2.67 | 0.05 | 3.41 | 0.23 | 3.99 | 0.13 | 4.81 | 0.23 |
| FL512397 | 1.00E-16 | Put ABA responsive protein | 2.32 | 0.05 | 2.15 | 0.29 | 3.60 | 0.09 | 1.22 | 0.06 |
| FL512396 | 1.00E-42 | Salt tolerant protein | 2.11 | 0.03 | 1.66 | 0.12 | 3.44 | 0.10 | 1.54 | 0.03 |
| FL518936 | 4.00E-17 | Salt tolerance protein 4 | 2.03 | 0.29 | 2.50 | 0.14 | 3.00 | 0.14 | 2.23 | 0.18 |
| FL512411 | 2.00E-40 | SAP | 2.12 | 0.10 | 1.63 | 0.20 | 3.21 | 0.18 | 1.18 | 0.11 |
