# Supplementary material for: Analysis of gene expression in response to water deficit of chickpea (Cicer arietinum L.) varieties differing in drought tolerance
Source: BMC Plant Biol. 2010 Feb 9;10:24. doi: 10.1186/1471-2229-10-24 (PMC2831037; doi:10.1186/1471-2229-10-24)
Supplement: Additional file 5 — Figure showing detail expression profiles of ESTs within each cluster made by SOTA clustering of fold expression of 53 high expressing ESTs in PUSABGD72. [file 1471-2229-10-24-S5.PPT]

## Slide 1
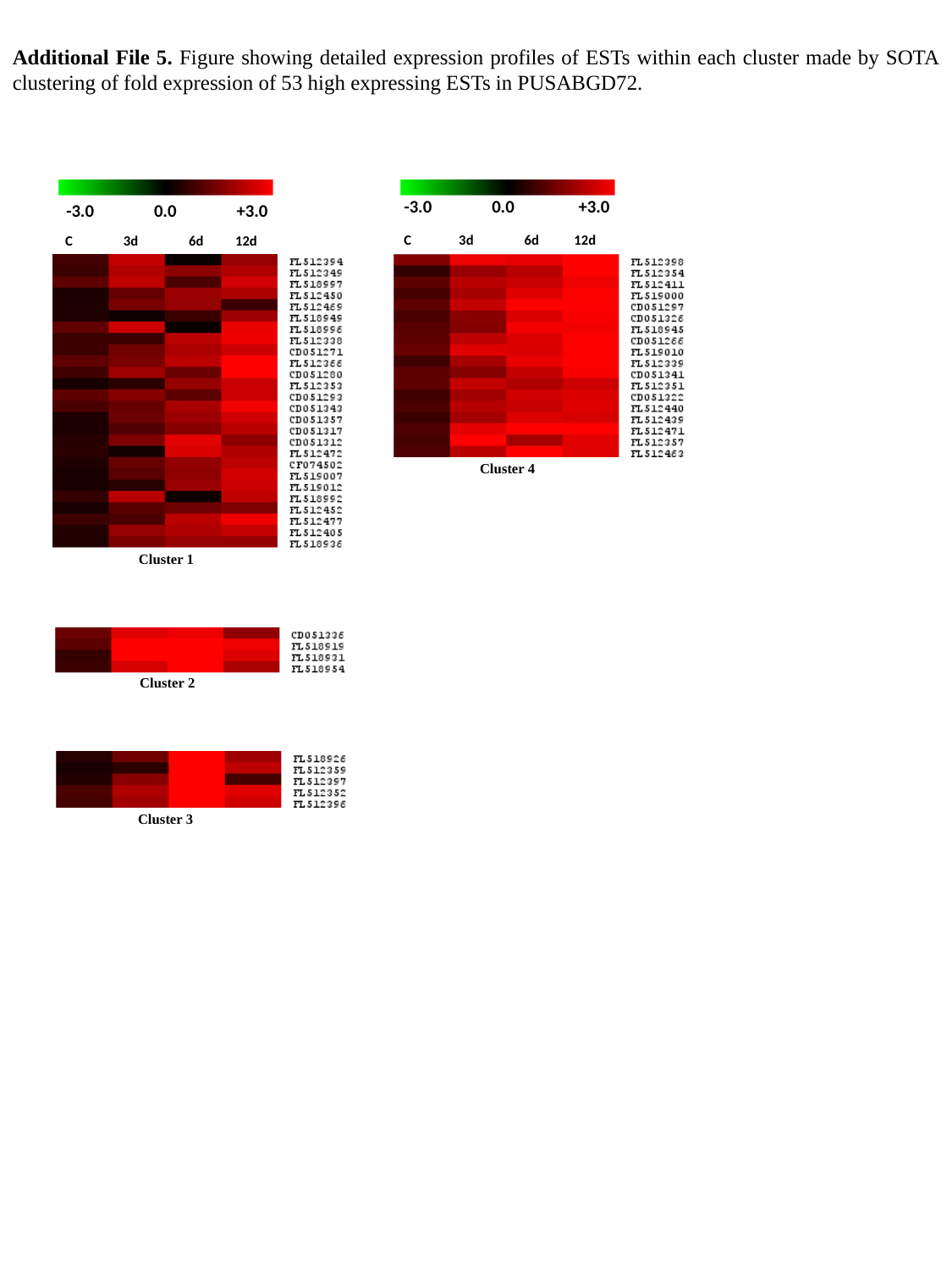

Additional File 5. Figure showing detailed expression profiles of ESTs within each cluster made by SOTA clustering of fold expression of 53 high expressing ESTs in PUSABGD72.
-3.0 0.0 +3.0
-3.0 0.0 +3.0
C 3d 6d 12d
C 3d 6d 12d
Cluster 4
Cluster 1
Cluster 2
Cluster 3
