# Supplementary material for: Analysis of gene expression in response to water deficit of chickpea (Cicer arietinum L.) varieties differing in drought tolerance
Source: BMC Plant Biol. 2010 Feb 9;10:24. doi: 10.1186/1471-2229-10-24 (PMC2831037; doi:10.1186/1471-2229-10-24)
Supplement: Additional file 6 — Table showing detail clustering of 53 high expressing ESTs according to their expression pattern in PUSABGD72. [file 1471-2229-10-24-S6.DOC]

**Additional File 6**: Table showing detailed clustering of 53 high expressing ESTs according to their expression pattern in PUSABGD72.

| **Annotation** | **Classification** | **Acc. No.** | **Log_2_(ctrl)** | **Log_2_ (3d)** | **Log_2_ (6d)** | **Log_2_ (12d)** |
| --- | --- | --- | --- | --- | --- | --- |
| **Cluster 1** | | | | | | |
| Class 10 PR protein | Cell Defence | FL512394 | 0.804 | 2.304 | 0.114 | 1.792 |
| MRP like ABC transporter | Cell Transport | FL512349 | 0.716 | 2.100 | 1.670 | 2.080 |
| MRP like ABC transporter | Cell Transport | FL518997 | 1.102 | 2.261 | 0.901 | 2.487 |
| Put mem protein | Cellular Organization | FL512450 | 0.363 | 1.179 | 1.838 | 2.028 |
| Non specific lipid transfer | Cellular Organization | FL512469 | 0.354 | 1.445 | 1.771 | 0.706 |
| Cellulase synthase | Cellular Organization | FL518949 | 0.383 | 0.184 | 0.706 | 1.854 |
| HSP 70 cognate | Cellular Organization | FL518996 | 1.145 | 2.414 | 0.142 | 2.780 |
| Metallothionein | Cellular Organization | FL512338 | 0.729 | 0.705 | 2.220 | 2.816 |
| LEA-1 | Cellular Organization | CD051271 | 0.724 | 1.366 | 2.035 | 2.434 |
| Cu/Zn superoxide dismutase II | Energy metabolism | FL512366 | 1.132 | 1.443 | 2.222 | 3.108 |
| P type H^+^ATPase | Energy metabolism | CD051280 | 0.770 | 1.874 | 1.251 | 3.241 |
| Allantoinase | Metabolism | FL512353 | 0.282 | 0.536 | 1.773 | 2.369 |
| Ubiquitin conjugating protein | Protein Degradation | CD051293 | 1.111 | 1.596 | 1.141 | 2.392 |
| Put protein kinase | Signal Transduction | CD051343 | 0.903 | 1.213 | 1.993 | 2.878 |
| Jasmonic acid 2 | Signal Transduction | CD051357 | 0.356 | 1.275 | 1.826 | 2.451 |
| Protein kinase | Signal Transduction | CD051317 | 0.352 | 0.974 | 1.609 | 2.171 |
| Protein phosphatase 2C | Signal Transduction | CD051312 | 0.460 | 1.503 | 2.684 | 1.688 |
| CBL-interacting protein kinase | Signal Transduction | FL512472 | 0.516 | 0.245 | 2.573 | 2.072 |
| AP2 domain like protein | Transcription | CF074502 | 0.385 | 1.257 | 1.738 | 2.247 |
| Put AP2 domain transcriptional regulator | Transcription | FL519007 | 0.310 | 1.059 | 1.667 | 2.499 |
| Transcriptional repressor of GlcNag | Transcription | FL519012 | 0.307 | 0.484 | 1.823 | 2.432 |
| α-NAC | Transcription | FL518992 | 0.623 | 2.189 | 0.188 | 2.273 |
| 60S ribosomal protein L27A | Translation | FL512452 | 0.310 | 1.059 | 1.303 | 1.506 |
| Put. Leunig | Unclassified | FL512477 | 0.707 | 0.883 | 2.223 | 2.831 |
| Put. Proline rich protein | Unclassified | FL512405 | 0.427 | 1.766 | 2.068 | 2.340 |
| Salt tolerance protein 4 | Unclassified | FL518936 | 0.410 | 1.460 | 1.766 | 1.781 |
| **Cluster 2** | | | | | | |
| Cysteine proteinase type protein | Protein Degradation | CD051336 | 1.281 | 2.637 | 2.802 | 1.713 |
| Elongation factor 1 alpha | Translation | FL518919 | 1.072 | 5.755 | 4.492 | 2.814 |
| Ribosomal protein L18a | Translation | FL518931 | 0.642 | 3.659 | 3.381 | 2.602 |
| Ribosome associated protein p40 | Translation | FL518954 | 0.714 | 2.527 | 3.385 | 2.013 |
| **Cluster 3** | | | | | | |
| Nodule enhanced sucrose synthase | Metabolism | FL518926 | 0.459 | 1.311 | 3.171 | 1.874 |
| Put. RNA bp | Transcription | FL512359 | 0.300 | 0.514 | 4.650 | 2.227 |
| Put ABA responsive protein | Unclassified | FL512397 | 0.414 | 1.622 | 3.188 | 0.846 |
| Proline rich protein | Unclassified | FL512352 | 0.900 | 2.070 | 4.511 | 2.667 |
| Salt tolerant protein | Unclassified | FL512396 | 0.834 | 1.897 | 3.106 | 2.449 |
| **Cluster 4** | | | | | | |
| Disease resistance response protein | Cell Defence | FL512398 | 1.535 | 2.792 | 2.737 | 3.364 |
| Aquaporin like Water channel protein | Cell Transport | FL512354 | 0.609 | 1.798 | 2.169 | 3.954 |
| SAP | Cellular Organization | FL512411 | 1.105 | 2.175 | 2.355 | 2.809 |
| Imbibition protein | Cellular Organization | FL519000 | 0.847 | 1.984 | 2.627 | 2.991 |
| LEA protein 2 | Cellular Organization | CD051326 | 0.873 | 1.630 | 2.560 | 2.954 |
| Nucleotide sugar epimerase like protein | Metabolism | FL518945 | 1.050 | 1.571 | 2.894 | 2.873 |
| β-amylase | Metabolism | CD051266 | 1.111 | 2.242 | 2.617 | 3.365 |
| Put β-amylase | Metabolism | FL519010 | 1.257 | 2.668 | 2.590 | 3.368 |
| Early leaf senescence abundant cysteine proteinase | Protein Degradation | FL512339 | 0.761 | 1.950 | 2.742 | 3.128 |
| ATP dependent clp protease | Protein Degradation | CD051341 | 1.111 | 1.586 | 2.313 | 2.940 |
| 14-3-3 brain protein homolog | Signal Transduction | FL512351 | 1.136 | 2.331 | 2.078 | 2.418 |
| G-protein coupled receptor like protein | Signal Transduction | CD051322 | 0.766 | 1.891 | 2.462 | 2.591 |
| SOS2 like protein | Signal Transduction | FL512440 | 0.911 | 2.156 | 2.333 | 2.633 |
| Zn finger protein | Transcription | FL512439 | 0.718 | 1.938 | 2.601 | 2.545 |
| Dehydration responsive element bp3 | Transcription | FL512463 | 0.911 | 2.192 | 3.071 | 2.644 |
| Dehydration induced protein | Unclassified | FL512471 | 0.925 | 2.694 | 3.209 | 3.252 |
| Leu rich recepter like protein | Unclassified | FL512357 | 0.824 | 3.479 | 1.964 | 2.657 |
