# Supplementary material for: Analysis of gene expression in response to water deficit of chickpea (Cicer arietinum L.) varieties differing in drought tolerance
Source: BMC Plant Biol. 2010 Feb 9;10:24. doi: 10.1186/1471-2229-10-24 (PMC2831037; doi:10.1186/1471-2229-10-24)
Supplement: Additional file 7 — Quantitative Real time-PCR analysis in different biological samples of PUSABGD72 and ICCV2 showing normalized fold induction of two ESTs (FL12394, FL518992) upon drought treatment. The experiment was done in triplicates by taking Actin as an internal control. [file 1471-2229-10-24-S7.PPT]

## Slide 1
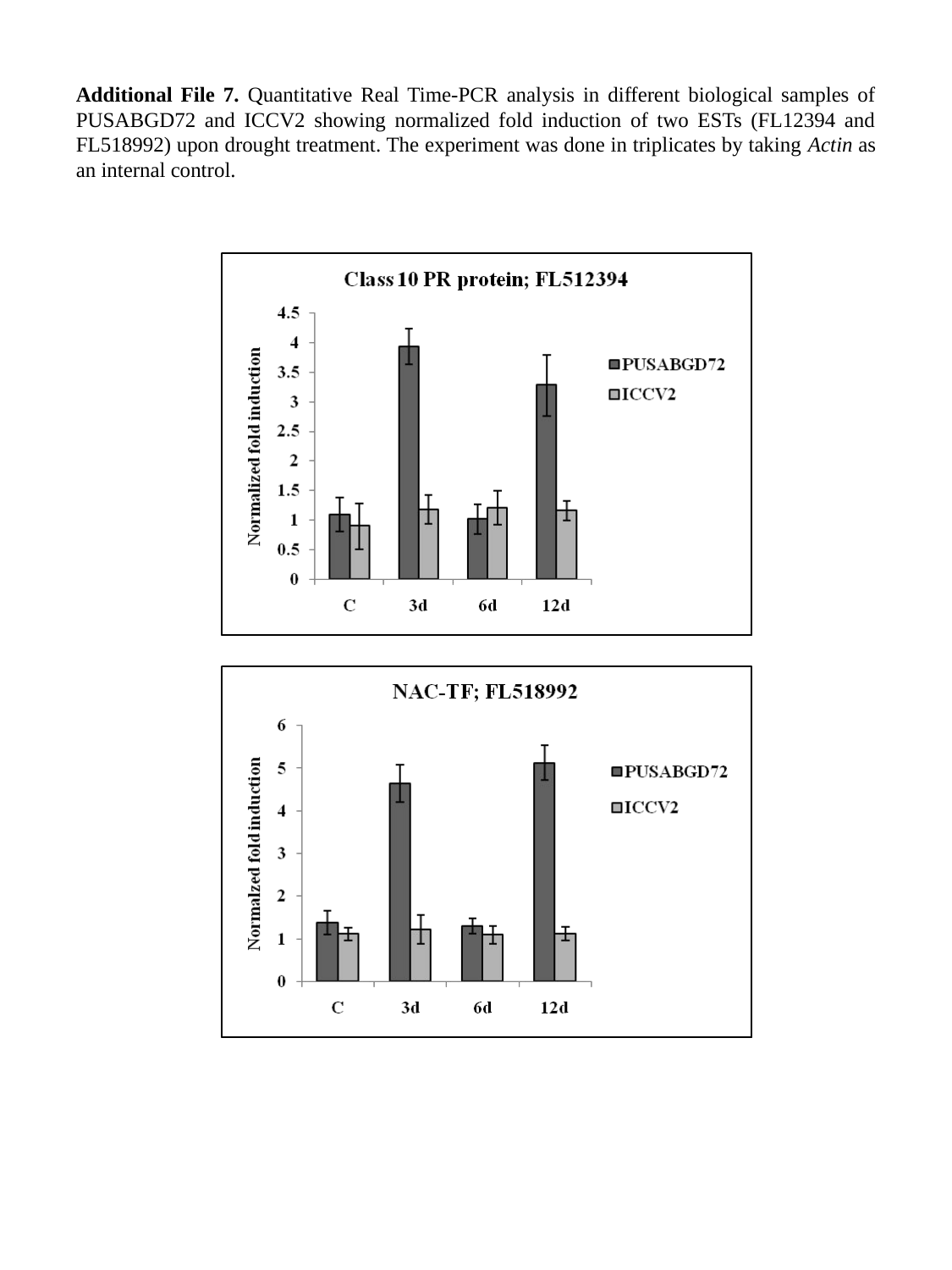

Additional File 7. Quantitative Real Time-PCR analysis in different biological samples of PUSABGD72 and ICCV2 showing normalized fold induction of two ESTs (FL12394 and FL518992) upon drought treatment. The experiment was done in triplicates by taking Actin as an internal control.
